# Material Discovery and High Throughput Exploration of Ru Based Catalysts for Low Temperature Ammonia Decomposition

**DOI:** 10.3390/ma13081869

**Published:** 2020-04-16

**Authors:** Katherine McCullough, Pei-Hua Chiang, Juan D. Jimenez, Jochen A. Lauterbach

**Affiliations:** Department of Chemical Engineering, University of South Carolina, Columbia, SC 29208, USA; mccullke@email.sc.edu (K.M.); pchiang@email.sc.edu (P.-H.C.); jiminezj@email.sc.edu (J.D.J.)

**Keywords:** ammonia decomposition, high throughput, hydrogen production, ruthenium catalyst, promoter, low temperature

## Abstract

High throughput experimentation has the capability to generate massive, multidimensional datasets, allowing for the discovery of novel catalytic materials. Here, we show the synthesis and catalytic screening of over 100 unique Ru-Metal-K based bimetallic catalysts for low temperature ammonia decomposition, with a Ru loading between 1–3 wt% Ru and a fixed K loading of 12 wt% K, supported on γ-Al_2_O_3_. Bimetallic catalysts containing Sc, Sr, Hf, Y, Mg, Zr, Ta, or Ca in addition to Ru were found to have excellent ammonia decomposition activity when compared to state-of-the-art catalysts in literature. Furthermore, the Ru content could be reduced to 1 wt% Ru, a factor of four decrease, with the addition of Sr, Y, Zr, or Hf, where these secondary metals have not been previously explored for ammonia decomposition. The bimetallic interactions between Ru and the secondary metal, specifically RuSrK and RuFeK, were investigated in detail to elucidate the reaction kinetics and surface properties of both high and low performing catalysts. The RuSrK catalyst had a turnover frequency of 1.78 s^−1^, while RuFeK had a turnover frequency of only 0.28 s^−1^ under identical operating conditions. Based on their apparent activation energies and number of surface sites, the RuSrK had a factor of two lower activation energy than the RuFeK, while also possessing an equivalent number of surface sites, which suggests that the Sr promotes ammonia decomposition in the presence of Ru by modifying the active sites of Ru.

## 1. Introduction

Ammonia has proven to be a promising CO_x_-free candidate for hydrogen storage and transportation [1,2]. The main advantages of using ammonia as a hydrogen storage material are its relatively high energy density, existing infrastructure, hydrogen storage capacity, and ability to be liquefied at 293 K and 8 atm of pressure for transportation [1]. Solid hydrogen storage materials, such as metal hydrides and metal organic frameworks, are an attractive alternative, but the release of H_2_ from these materials requires harsh operating conditions and often suffers from poor H_2_ sorption reversibility [1]. Additionally, these materials have lower hydrogen storage capabilities than ammonia. For example, 10 kg of H_2_ can be stored in either 96 kg of liquid ammonia (108 g_H2_/L), 392 kg of Mg_2_NiH_4_ (40 g_H2_/L), or 730 kg of LaNi_5_H_6_ (36 g_H2_/L) [1,3].

Despite the obvious advantages of H_2_ storage through ammonia, on-site power generation via hydrogen fuel cells requires the catalytic decomposition of ammonia, and thus limited by the performance of the catalytic material. This process requires a highly active ammonia decomposition catalyst that can generate H_2_ from ammonia at moderate to low temperatures (below 450 °C) in order to avoid poisoning or degradation of the hydrogen separation membranes, which often exhibit low thermal stability [4]. Supported Ru catalysts are regarded as the most active ammonia decomposition metal and have been studied for over a century. Attempts to utilize lower cost alternatives, such as Fe [5,6,7,8], Co [5,9,10,11], Ni [12,13,14,15,16,17], Cr [18], and Cu [19] have been demonstrated, but they fail to reach appreciable activity at low temperatures. Their lower apparent activity is attributed to the N_2_ binding energy of these metals, as they either bind N_2_ too strongly or too weakly to facilitate ammonia decomposition. It is generally accepted that the recombinative desorption of N_2_ is the rate determining step for ammonia decomposition [20,21,22,23].

Modifications outside the realm of current promotional enhancement is required in order to find a low cost, low temperature ammonia decomposition catalyst. A number of studies continue to focus on the optimization of Ru based systems. This is typically achieved by providing electronic modification with the addition of alkali and alkaline earth metals, specifically K, Cs, and Ba [24,25,26,27,28], or by manipulating the dispersion and size of Ru particles [29,30,31], thus taking advantage of the structure sensitive nature of the reaction. Additionally, using highly basic support materials provides further electronic modification to the catalyst [32,33,34,35,36]. Intelligent design and theoretical investigations of novel bimetallic catalysts for ammonia decomposition are most frequently based on calculated N_2_ binding energies. Thus, bimetallic catalysts have been created by taking one metal with a higher binding energy than Ru and another with lower binding energy, so that the linear combination of the two produces a catalyst with a similar binding energy to Ru. This has been demonstrated with Co-Mo [37,38,39], Fe-Co [40] and Ni-Fe [41] alloys and bimetallic catalysts. However, these bimetallic catalysts are not able to achieve activity within the low temperature ranges that are needed for PEM fuel cells and were significantly outperformed by Ru based catalysts. 

Being motivated by the lack of compositional diversity in this field, we sought to perform a comprehensive screening for ammonia decomposition catalysts with the aim to discover new catalyst compositions, reduce Ru content, and obtain near thermodynamic ammonia conversion at temperatures at or below 400 °C under realistic operating conditions. A catalyst containing 4 wt% Ru promoted by 12 wt% K supported on gamma-alumina was used as a base catalyst composition, and was systematically modified by substituting Ru with different alkaline earth metals, transition metals, noble metals, and metalloids. These include Mg, Ca, Sr, Sc, Y, Zr, Hf, Nb, Ta, Cr, Mo, W, Mn, Re, Fe, Os, Co, Rh, Ir, Ni, Pd, Pt Cu, Ag, Au, Zn, Cd, In, Sn, Pb, and Bi. In this way, we were able to study trends in activity as the ratio of Ru metal to the substituted metal decreased from 3:1 to 2:2 to 1:3. In total, 33 different metals and 94 unique catalyst formulations were synthesized and then tested for their low temperature ammonia decomposition activity using a 16-channel parallel high throughput reactor system. Catalysts containing Hf, Y, Sc, Sr, Mg, Zr, Ta, and Ca were able to achieve high activity with less Ru. Additionally, the Sr, Y, Zr, and Hf catalysts were able to achieve greater than 80% conversion at 400 °C with as little as 1% Ru. To the best of our knowledge, these catalyst materials have not been previously reported. In this work, we investigated the kinetics and adsorption properties of these Ru based Sr and Fe containing catalysts to understand the effect that secondary metal substitution has on activity.

## 2. Materials and Methods 

### 2.1. Catalyst Synthesis

The catalysts were synthesized using the incipient wetness impregnation technique and were supported on SBA-200 γ-Al_2_O_3_ (Catalox, 99.99%, 192 ± 20 m^2^/g, 30 Å pore radius, 0.45 mL/g pore volume), where the support materials was used as received from the manufacturer. KCH_3_COO (Fischer Scientific, Waltham, MA, USA, 98% purity), anhydrous RuCl_3_, and anhydrous chloride salts of the following: Mg, Ca, Sr, Sc, Y, Zr, Hf, Nb, Ta, Cr, Mo, W, Mn, Re, Fe, Os, Co, Rh, Ir Ni, Pd, Pt, Cu, Ag, Au, Zn, Cd, In, Sn, Pb, and Bi (Sigma Aldrich, ≥ 95% purity) were used without further modification. The support was first dried at 120 °C for 2 h before impregnation. An appropriate amount of RuCl_3_, secondary anhydrous chloride salt, and KCH_3_COO were mixed to obtain 3:1:12, 2:2:12, or 1:3:12 weight ratios of Ru:M:K (where M = Mg, Ca, Sr, Sc, Y, Zr, Hf, Nb, Ta, Cr, Mo, W, Mn, Re, Fe, Os, Co, Rh, Ir Ni, Pd, Pt Cu, Ag, Au, Zn, Cd, In, Sn, Pb, or Bi) and dissolved in DI water. An aliquot of solution was then added to the dried support under magnetic stirring until incipient wetness was achieved. The impregnated support was then dried at 120 °C for 30 min., after which the process was repeated until the entire solution was impregnated onto the support. The catalysts were then ground and heated at 200 °C for 2 h, and subsequently calcined in air at 550 °C for 3 h. 

### 2.2. Catalytic Performance 

#### 2.2.1. High-Throughput Screening

Catalytic testing was carried out in a 16-channel parallel reactor system. The temperature of each of each of the 16 catalyst beds was measured using K-type thermocouples. The gas effluent of the reactor system was analyzed using a Bruker Equinox 55 FT-IR spectrometer (Bruker, Billerica, MA, USA) coupled with a 128 × 128-pixel mercury cadmium telluride (MCT) focal plane array. Further details of the system along with a reactor schematic can be found elsewhere [42,43]. Various known concentrations of NH_3_ were flown through the empty reactor channels and their IR spectra were acquired for each reactor channel in order to quantify the effluent stream. The integrated peak area was used to correlate the IR signal with NH_3_ concentration using GRAMS AI software (version 9.3, ThermoFischer, Waltham, MA, USA). The catalysts were first heated to 450 °C under Ar followed by a reduction in 10% H_2_ for 1 h. Catalyst activity was screened while using 200 mg of catalyst and at a space velocity of 30,000 mL/hr/g_cat_ under 1% NH_3_ in balance Ar and at atmospheric pressure. Measurements were taken in 50 °C increments from 250 °C to 400 °C.

#### 2.2.2. Catalyst Activity under Pure Ammonia

The catalysts were run under pure NH_3_ (99.995%, Airgas) in order to measure catalytic activity, activation energies under differential conditions (2–12% conversion) and the turnover frequency (TOF) at 300 °C, 350 °C and 400 °C. A single channel plug flow reactor was used where the catalysts was supported in the center of the furnace using quartz wool and a supporting rod. The flowrates were controlled using Brooks Mass Flow Controllers (Brooks Instrument, Hatfield, PA, USA). Catalysts were first heated to 450 °C in Ar and then subjected to a 10% H_2_ reduction for one hour. For catalyst activity, NH_3_ conversion was then measured in 50 °C increments from 250 °C to 400 °C. The catalyst bed temperature was measured using a K-type thermocouple and the space velocity was kept constant for each reaction at 5400 ml_NH_3__hr^−1^g-cat^−1^ using 500 mg of catalyst. The thermodynamic equilibrium calculations for ammonia decomposition at 1 bar may be found in Figure S1. The product stream was analyzed using a Shimadzu 2014 gas chromatogram (Shimadzu, Kyoto, Japan) that was equipped with a thermal conductivity detector (TCD). The GC is equipped with a Mol Sieve 5A plot column for H_2_ and N_2_ separation. The concentration of H_2_ and N_2_ in the product stream were used to calculate the NH_3_ conversion at each temperature and they were always found to be in stoichiometric proportions. NH_3_ conversion using pure γ-Al_2_O_3_ was first measured under these conditions and found to be negligible at all temperatures. The activation energy measurements were carried out twice for each catalyst, and the average activation energy and standard deviation is reported.

### 2.3. Catalyst Characterization

X-ray diffraction (XRD) was carried out to determine the structural composition of the active catalyst components. XRD was carried out for all of the samples in a Rigaku Miniflex II (Tokyo, Japan) that was equipped with a Cu-Kα X-ray source and a high-speed silicon-strip detector. The scans were completed between a 10 and 80° 2θ angle at a rate of 2°/min. with step size of 0.02°. Hydrogen chemisorption was conducted on a Micromeritics Autochem II 2920 (Micromeritics, Norcross, GA, USA) equipped with a TCD, in order to determine the number of exposed Ru surface atoms per gram of Ru. Catalysts were first reduced at 450 °C in 10% H_2_/Ar and then heated to 460 °C for 15 min under inert gas to remove excess H_2_ from the surface. The catalysts were then cooled to 50 °C, and a 10% H_2_/Ar mixture was pulsed, and the amount of H_2_ adsorbed was determined. The turnover frequencies were calculated assuming a H_2_:Ru ratio of 1:1 [44]. CO adsorption was performed via diffuse reflectance IR spectroscopy while using a Bruker Vertex 70 FT-IR spectrometer (Bruker, Billerica, MA, USA) equipped with an MCT detector cooled by liquid nitrogen and a Praying Mantis Diffuse Reflectance cell (Harrick Scientific Products, Pleasantville, NY, USA). The spectra were taken with a resolution of 4 cm^−1^ and averaged over 512 scans. The catalysts were first heated to 450 °C under N_2_ (UHP, Airgas), and then subjected to a 10% H_2_ pretreatment for one hour. The catalysts were then cooled to 50 °C under inert, and a mixture gas of 1000 ppm CO in balance He was used for room temperature CO adsorption. The SEM images were taken on a Zeiss Ultra Plus FESEM (Carl Zeiss, Oberkochen, Germany) and TEM images were taken on a Hitachi HT7800 TEM (Hitachi, Tokyo, Japan). 

## 3. Results and Discussion

### 3.1. Design Space for the Initial High Throughput Screening

Previous work from our group utilized a response surface methodology to optimize the promoter elements and weight loading for a Ru based catalyst for ammonia decomposition, where the optimal promoter element and loading for 4 wt% Ru/γ-Al_2_O_3_ was found to be 12 wt% K [45]. The resultant 4%Ru-12%K/γ-Al_2_O_3_ catalyst was chosen as the starting point for our initial high throughput study. The Ru content was then reduced and substituted with different elements in order to determine to what extent the replacement of Ru with a secondary element can be modified. The total metal weight loading was kept constant at 4% for all catalysts in order to isolate the influence of the secondary element. The Ru content was reduced by either 1%, 2%, or 3% and substituted with either Mg, Ca, Sr, Sc, Y, Zr, Hf, Nb, Ta, Cr, Mo, W, Mn, Re, Fe, Os, Co, Rh, Ir, Ni, Pd, Pt, Cu, Ag, Au, Zn, Cd, In, Sn, Pb, or Bi, so that the total metal weight loading sums to 4%. This resulted in three different catalyst formulations for each of the 31 elements studied. The ratio of Ru metal to secondary metal was 3:1, 2:2, or 1:3. Each catalyst was promoted with 12% K and supported on γ-Al_2_O_3_. For example, a catalyst containing 3% Ru, 1% Fe, and 12%K, and supported on γ-Al_2_O_3_ will be referred to as 3,1,12 RuFeK, and this nomenclature will be used for all catalysts and weight loadings.

It is worth noting that the K promotion and weight loading used will not be at an optimum for all Ru substituted catalysts studied. However, we can ensure that changes in reaction order will be due to the substituted secondary metal by using the same promoter/support combination throughout the study, and not due to newly introduced promoter support interactions, which is beyond the scope of this work. Additionally, because the support material is also kept constant, we can ensure that the interaction between the promoter and the support material is consistent across each catalyst, as different promoters will exhibit different modification effects, depending on the support used [26,46,47]. The same support utilized in the original response surface study mentioned previously was applied herein because of the dependency of the support on the effect of promoter modification.

#### Characterization of 4 Ru/Al_2_O_3_ and 4,12 RuK/Al_2_O_3_ Baseline Catalysts

**Figure 1:**
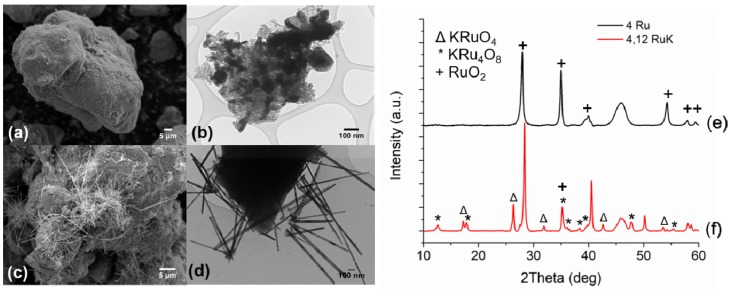
Left panel: SEM images of (**a**) 4 Ru/Al_2_O_3_ and (**c**) 4,12 RuK/Al_2_O_3_. TEM images of (**b**) 4 Ru/Al_2_O_3_ and (**d**) 4,12 RuK /Al_2_O_3_. Right panel: corresponding XRD patterns of (**e**) 4 Ru/Al_2_O_3_ and (**f**) 4,12 Ru/Al_2_O_3_.

A baseline catalyst containing 4 wt% Ru and 12% K supported on γ-Al_2_O_3_ was first synthesized in order to make effective comparisons of the substituted Ru catalyst, as described elsewhere [45]. Figure 1a,b show the SEM and TEM image of the unpromoted baseline catalyst and Figure 1c,d show the SEM and TEM image of the 4,12 RuK catalyst. It is apparent from the SEM and TEM analysis that the addition of K influences the Ru morphology on the catalyst surface, which correlates to the KRu_4_O_8_ hollandite structure [45]. The hollandite structure is a one-dimensional material that consists of edge sharing RuO_6_ octahedrons that share corner oxygens with adjoining octahedrons, thus forming 2 × 2 square tunnels, which contain K cations [48,49,50]. This structure can be observed in both the SEM and TEM images of the 4,12 RuK catalyst. Figure 1e,f show the XRD patterns for the unpromoted 4 wt% Ru catalyst (4 Ru) and the baseline K promoted Ru catalyst (4,12 RuK).

In both XRD patterns, amorphous Al_2_O_3_ is apparent at around 46°. The unpromoted 4 wt% Ru catalysts forms large RuO_2_ domains, corresponding to reflections positioned at 28°, 35°, 40°, 57.9°, and 59.4° [51,52]. The size of these domains, calculated via Scherrer’s Equation, is roughly 23.1 nm. The 4,12 RuK includes several additional reflections. First, crystalline KCl is apparent at 28.33°, 40.5°, 50.1°, and 58.6° (not labeled for clarity). The slight shoulder on the left of the KCl reflection at 28° indicates that there are small amounts of RuO_2_ present in the 4,12 RuK catalyst. New phases appear at 17.2°, 26.3°, 42.5°, and 53.5° that correspond to KRuO_4_ [53,54], with an average domain size of 29.8 nm. Additionally, the reflections that appear at 12.6°, 17.8°, and 35.1° correspond to the hollandite phase KRu_4_O_8_ [48,54], with an average crystallite size of 17.8 nm. 

The catalysts with the Ru based hollandite structure present before reaction were previously found to have increased low temperature ammonia decomposition activity [30], although polycrystalline hollandite and its use as a catalytic material is not well understood. Here, Ru is found in the 3^+^/4^+^ oxidation state. One reason for the increased activity of KRu_4_O_8_ over RuO_2_ nanoparticles might be due to the intimate positioning of K and Ru in these structures. In this way, the catalyst shows increased activity due to electron donation. Additionally, KRu_4_O_8_ can act as a precursor for disordered Ru^0^ structures, which result in in the formation of crystal defects that could greatly enhance the intrinsic activity of Ru based catalysts, where an increase in the defect density on Ru has been shown to greatly improve catalytic performance [22,55]. 

### 3.2. High-Throughput Screening of Ru Based Catalysts

**Figure 2:**
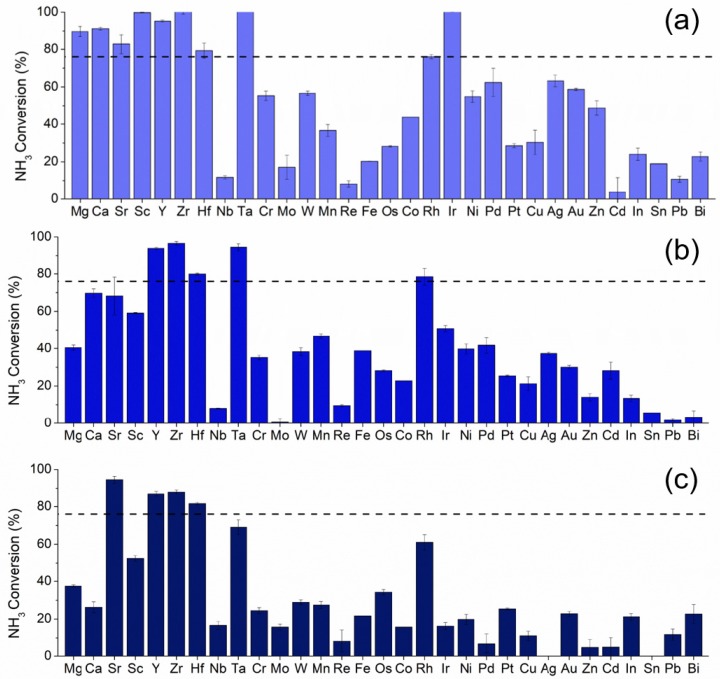
Catalytic activity at 300 °C of (**a**) 3,1,12 RuMK, (**b**) 2,2,12 RuMK, and (**c**) 1,3,12 RuMK, where the secondary metal M, is listed on the x-axis. The black dashed line indicates the activity of the baseline 4,12 RuK catalyst at 300 °C. Reaction conditions: 1% NH_3_/Ar, 1.01 bar, 30,000 mL/hr/g_cat._

The K promoted Ru based catalysts with metal substitution were screened for ammonia decomposition activity in 1% NH_3_/Ar at 30,000 mL/hr/g_cat_, where Figure 2 shows the results of the screen at 300 °C. The horizontal dashed line corresponds to the activity of the baseline 4,12 RuK catalyst. Figures S2–S4 show the catalytic activity of all compositions at 250 °C, 350 °C, and 400 °C. Catalytic performance at 300 °C was used to determine the successful catalysts from the screen, due to the large range of responses exhibited, and in the interest of finding substitutional materials that exhibit low temperature activity. Catalyst compositions exceeding the activity of the promoted baseline catalyst (4,12 RuK) are considered to be successful. Due to the complex relationship present in these catalysts, a general discussion of trends will be given herein, followed by a more in-depth comparison between the Sr and Fe containing catalysts.

The crystalline structure of the entire catalyst library was probed via XRD in order to determine the phases of Ru present and whether secondary phase formation occurred with the addition of a secondary metal. The complete XRD analysis of all the materials can be found in the Supplemental Information (Figures S6–S32). Catalysts containing 3% Ru (Figure 2a) and 1% of Mg, Sr, Ca, Hf, Sc, Ta, Zr, Ir, or Y achieved greater than 83% conversion at 300 °C. As the loading of Ru decreased to 2% (Figure 2b), the activity of Mg, Sc, and Ca containing catalysts declined, while Y, Zr, Sr, Hf, and now Rh, continued to exhibit high performance. With the further decrease to 1% Ru (Figure 2c), only Sr, Y, Zr, and Hf substitution continued to remain highly active. In contrast, Bi, In, Mo, Nb, Cu, Re, Cd, Sn, and Pb lowered the catalytic performance, independent of the Ru loading. This might be due to the poor electronegativity and electron withdrawing nature of these elements, as previous studies have shown that dopants and supports with strong basicity and electrical conductance greatly enhance activity [56,57,58,59,60]. For example, small amounts of Nb have been shown to drastically increase the number of Lewis acid sites present in bulk Ni-Nb-O mixed metal oxides, and thus the enhanced acidity may be responsible for the poor activity seen here [61]. 

While catalysts containing 3% Ru and 1% Mg, Sr, Ca, Sc, or Y outperformed the baseline 4,12 RuK composition at 300 °C, as the ratio of Ru to secondary metal was further decreased, the activity of Mg, Sc, and Ca containing catalysts drastically declined, while the Y and Sr catalysts remained highly active with as low as 1% Ru. While alkali metals are regarded as electronic promoters, Mg and Ca are usually considered to be structural promoters [28,29]. Thus, while Mg and Ca may act to increase the number of active sites present on the catalyst, the intrinsic rate of these sites would not be modified [62]. Therefore, as the Ru loading decreases, the number of active sites present would also decrease with the addition of Mg and Ca.

The substitution of Ru with transition metals, primarily Group V–VII, generally resulted in a catalyst with poorer activity than the baseline catalyst. As the Ru loading continued to decrease from 3 wt% (Figure 2a) to 1 wt% (Figure 2c), there is an observed decline in activity that remains relatively constant, regardless of the catalyst composition. Ru active sites are being replaced with different sites that are intrinsically less active than Ru, as Ru is substituted with transition metals [23,37,63]. Therefore, the active sites of Ru are not being structurally or electronically modified, but simply replaced with those with lower rates of reaction or that hinder the rate determining step of the recombination and desorption of N_2_. The activity of the noble metal substituted catalysts showed a higher sensitivity to the variation in weight loadings, and a decrease in activity is observed with increasing noble metal loading. One reason for this observation might be due to the inhibitive effect of hydrogen on the reaction rate over Ru [26,64]. While small additions of noble metals can act as a promoter, larger relative loadings would thus result in a higher degree of inhibition due to the hydrogen spillover effect exhibited by these noble metals, such as Pt and Pd [65,66,67,68]. 

The activity of other monometallic catalysts for ammonia decomposition generally follows: Ru > Rh > Ni > Co > Ir > Fe >> Pt > Cr > Pd > Cu >> Pb [69]. The activity of the catalysts studied here do indeed follow the observed trend in activity of Rh > Ni > Co > Fe > Pt > Cr > Pd. Therefore, in general, we can conclude that the addition of Group V–VII transition metals ultimately did not result in any positive interactions between Ru and the substituted metal, due to the activity following the general trends of the monometallic metals that has already been established in the literature. It might then follow that the metals on the surface may be isolated from each other and not in intimate contact. Based on the summary of the crystalline structure and the catalytic performance, we are attributing the presence of KRuO_4_ and KRu_4_O_8_ to increased catalytic activity. However, we were not able to definitively correlate this claim due to the complexity of these structures and the difficulty of isolating these phases while also adding a secondary metal to the catalyst. Therefore, a detailed crystallographic study on the effects of phase on catalytic performance using *in situ* XRD and *in situ* electron microscopy would be of great interest, but outside the scope of this work.

**Figure 3:**
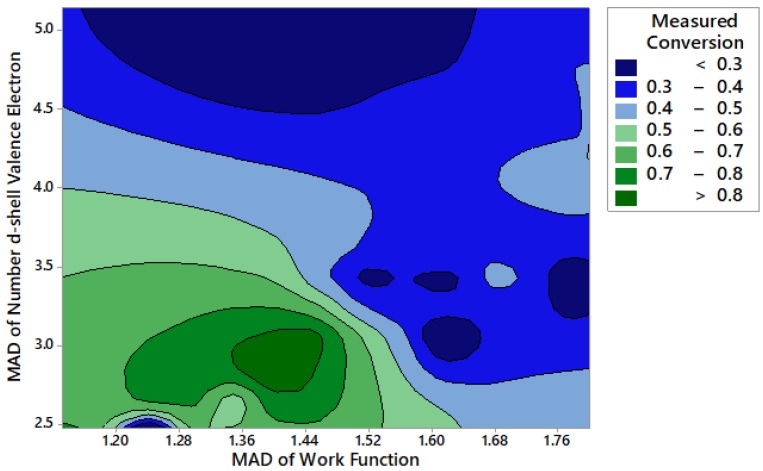
Ammonia decomposition activity at 300 °C as a function of the mean absolute deviation (MAD) of the number of d-shell valence electrons and the MAD of the catalyst work function.

Due to the complex relationships present in these multicomponent catalysts, a random forest machine learning algorithm was recently applied to a subset of the data shown here in order to determine what material properties led to catalysts with high activity [57]. While previous work has looked at single variables to describe the trends exhibited by metals for ammonia decomposition, no single variable has been able to correctly predict the trends across a variety of metals [63]. However, looking at two-way interactions between features provides new insight into the relationships between electronic characteristics and catalyst activity. For example, Figure 3 shows the relationship between activity and the mean absolute deviation (MAD) of the catalyst work function and the MAD of the number of d-shell valence electrons. It is apparent that catalysts with lower work function and a lower number of d-shell valence electrons resulted in a higher activity catalyst. 

In general, it was determined that the number of d-shell valence electrons, electronegativity, covalent radius, and work function all played an important role in determining the catalytic activity. These additional features are related to the electronic characteristics of the catalyst, where the electronic structure of the catalyst has been known to influence the strength of the bond between the metal surface and adsorbate, which affects the activity and reactivity of the catalyst [70,71]. Therefore, we further investigated the influence of highly active Sr and less active Fe catalysts on ammonia decomposition activity, and whether or not surface modification played an important role in activity.

### 3.3. Influence of Sr and Fe on NH_3_ Decomposition Acitivty and Kinetics

The addition of Sr to the Ru based catalysts showed that the Ru content could be reduced to 1% and still maintain high activity at 300 °C. While Mg, Ca, and Ba are often used in ammonia synthesis and decomposition for their excellent promotional abilities, there is little information on the other alkaline earth metal Sr, and its utilization in this reaction. Additionally, Fe is commonly used as an alternative to Ru for both ammonia synthesis and decomposition. However, we observed poor activity across all three weight loadings of Fe based catalysts. For these reasons, Sr and Fe were chosen to discern the trends in catalytic performance under higher ammonia feed gas concentrations to ultimately elucidate the reaction kinetics and surface properties of both high and low performing catalysts for ammonia decomposition. 

#### 3.3.1. Characterization of Sr and Fe Catalysts

**Figure 4:**
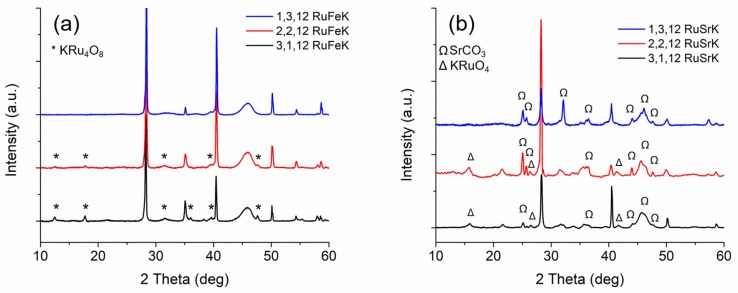
(**a**) XRD patterns of (from top to bottom): 1,3,12 RuFeK, 2,2,12 RuFeK and 3,1,12 RuFeK. (**b**) XRD patterns of (from top to bottom): 1,3,12 RuSrK, 2,2,12 RuSrK, and 3,1,12 RuSrK.

Figure 4a,b show the XRD patterns for each of each weight loading of the Fe and Sr catalysts, respectively. 

Fe containing catalysts contain sharp reflections at 28.33°, 40.5°, 50.1°, and 58.6°, which are indicative of KCl formation. The Fe catalysts primarily form crystalline KRu_4_O_8_, and decrease in relative intensity with decreased Ru loading, such that there are no Ru phases present in the 1,3,12 RuFeK, which indicates highly dispersed hollandite. No Fe phases were present, suggesting either the formation of amorphous Fe species or highly dispersed oxide species. In contrast, the Sr catalysts formed KRuO_4_ indicated by reflections at 17.2°, 26.3°, and 42.5° [53,54]. SrCO_3_ is present in all three Sr catalysts and increases in the relative intensity with decreasing Ru loading. Additionally, the relative intensity of KCl varied as the weight loading of Sr and Ru varied, which suggested that there might be more free Cl ions on the surface of the catalyst, or that an X-ray amorphous Sr oxo-chloro complex may have formed. Additionally, we observe strong K modification to the Al_2_O_3_ surface, which is evidenced by new reflections appearing at ca. 24.1° [72].

**Figure 5:**
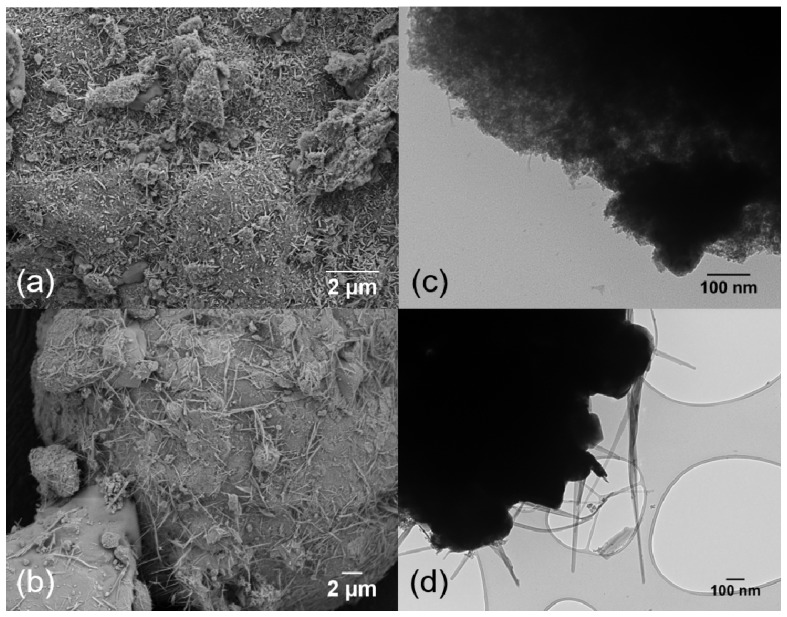
(**a**) SEM image and (**c**) TEM image of 1,3,12 RuFeK. (**b**) SEM image and (**d**) TEM images of 1,3,12 RuSrK.

Figure 5a shows a SEM image of the 1,3,12 RuFeK catalyst and Figure 5c shows the corresponding TEM image. Very small rod like particles are visible on the peripheral surface of the Al_2_O_3_, which confirm highly dispersed KRu_4_O_8_. Figure 5b,d show the SEM and TEM image of the 1,3,12 RuSrK, respectively. Figure 5d shows the KRuO_4_ complex, which exhibits a narrow, thin sheet-like structure, in contrast to the stiff rod-like structure that is exhibited by hollandite in Figure 1b. 

#### 3.3.2. Evaluation of Apparent Activation Energy and TOF

**Figure 6:**
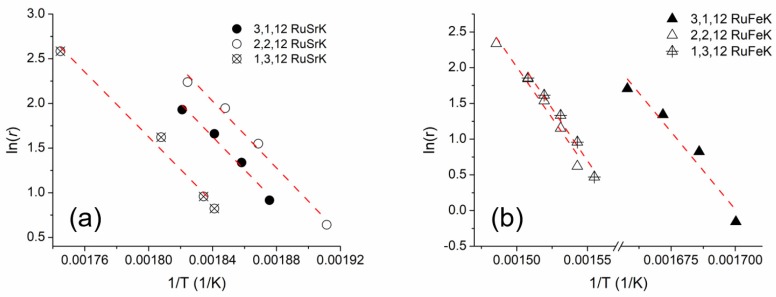
Arrhenius plot of (**a**) RuSrK and (**b**) RuFeK based catalysts. Reaction Conditions: 100% NH_3_, 5400 mL/hr/g_cat_ and 1 bar. Apparent activation energies were conducted under differential conditions.

The apparent activation energies were calculated for each of the three weight ratios of the Fe and Sr catalysts, for a total of six catalysts. The Arrhenius plots for are shown for the Sr and Fe in Figure 6a,b, respectively. The activation energies were calculated under differential conditions in 100% NH_3_ and at 5400 mL/hr/g_cat._ The Weisz–Prater criterion was used to determine the absence of internal mass transfer resistances [73,74] (see Supplemental Information), and no mass transfer limitations were observed. Figure S5 shows the Arrhenius plots for the 4 Ru and 4,12 RuK catalysts. 

Additionally, the turnover frequency (TOF) for each substituted Ru catalyst was determined and compared to the 4,12 RuK and unpromoted 4 Ru catalysts. The TOF was calculated by normalizing the rate of reaction to the number of exposed Ru atoms per gram of Ru, which were determined via H_2_ chemisorption. Table 1 provides the hydrogen uptake, TOF, and apparent activation energies for each catalyst. However, it should be noted that Fe in the presence of a more reducible metal, such as Ru, could result in Fe adsorbing H_2_ under the chemisorption conditions [75]. Therefore, the values reported for RuFeK based catalysts are actually an underestimation of the total number of sites, since a stoichiometry of 1:1 H_2_:Ru was used to calculate TOF.

With the addition of K to the 4 Ru catalyst, the apparent activation energy decreases from 125.2 kJ/mol to 65.7 kJ/mol and resulted in the suppression of H_2_ adsorption (at constant Ru wt%). Other apparent activations energies have been reported in a range from 87.9–155 kJ/mol [20,27,76,77] for supported Ru catalysts, which agrees with the results that are presented here. The decrease in apparent activation energy suggests electronic modification of the active sites, which has been thoroughly discussed in literature [34]. The Sr and Fe containing catalysts both exhibited a higher calculated apparent activation energy than the 4,12 RuK and 4 Ru catalyst, with the Sr catalysts within the range of 149.6–156.4 kJ/mol, and the Fe containing catalysts within the range of 226.6–250.9 kJ/mol. The apparent activation energy for the Fe containing catalysts is much higher than that calculated for the recombinative desorption of N_2_ on Ru (001) crystal, which was found to be roughly 184 kJ/mol [78]. Microkinetic modeling determined that the dehydrogenation of adsorbed NH_3_ and adsorbed NH_2_ yielded an activation energy of 43.9 and 65.3 kJ/mol, respectively, while the dehydrogenation of adsorbed NH has a much higher activation energy of 161.5 kJ/mol [64]. Therefore, the addition of Sr seems to change the rate limiting step from the recombinative adsorption of N_2_ on 4 Ru, to some combination of dehydrogenation of NH species on the surface, based on the apparent activation energies calculated here. This seems to be independent of the Ru and Sr weight loadings, since we do not observe a strong change in apparent activation energy with changes in the amount of Ru and Sr. The change in the rate limiting step on different metal surfaces has been previously confirmed [63]. However, little work has been reported regarding the influence of promoters. In contrast, for the Fe based catalyst, the change in the apparent activation energy is almost double that of the baseline 4 Ru catalyst, indicating a dramatic change in the active sites of the catalyst when Fe is co-added. 

**Figure 7:**
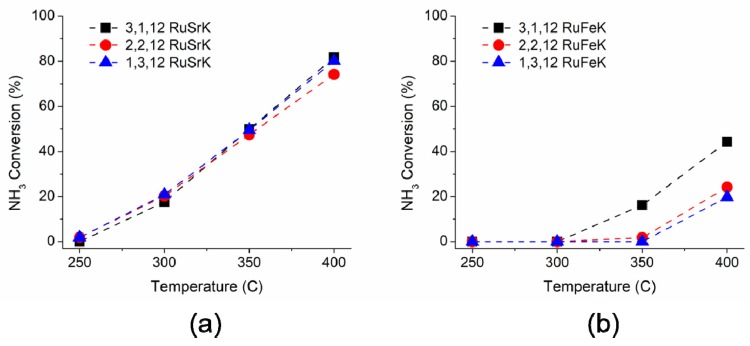
Ammonia decomposition activity of (**a**) 3,1,12 RuSrK, 2,2,12 RuSrK, and 1,3,12 RuSrK and of (**b**) 3,1,12 RuFeK, 2,2,12 RuFeK, and 1,3,12 RuFeK. Reaction conditions: 100% NH_3_, 1.01 bar, and 5400 mL/hr/g_cat_.

The ammonia decomposition activity of these catalysts was evaluated in a single channel reactor to further probe the catalytic performance beyond the capabilities of the high throughput reactor. Each catalyst was tested under 100% NH_3_ and 5400 mL/hr/g_cat._ The catalytic performance for ammonia decomposition is shown in Figure 7a,b for Sr and Fe, respectively. 

Upon increasing the ammonia concentration from 1% in the initial screen to 100%, all of the catalysts showed a decline in the rate of reaction at 300 °C when compared to the activity from the initial screen, as shown in Figure 2 (and at all other subsequent temperatures, as shown in Figure S2–S4). Previous studies have observed a similar negative dependence on the ammonia partial pressure [64]. For the Sr based catalysts, each of the three weight loadings exhibited similar activity in the range of 250–400 °C with the 3,1,12 RuSrK catalyst obtaining 82% conversion at 400 °C. When the weight loading of Ru was lowered to 1,3,12 RuSrK, the activity declined to only 80%. However, the 1,3,12 RuSrK exhibited the highest TOF of all catalysts studied of 1.78 s^−1^ at 400 °C, while the 3,1,12 RuSrK exhibited a TOF of 0.88 s^−1^ at 400 °C.

The Fe based catalysts showed a larger distribution of activity across the three weight loadings and showed no activity at 300 °C in contrast to the Sr catalysts. Additionally, the 1,3,12 RuFeK catalysts have a TOF of 0.28 s^−1^ at 400 °C, which was the lowest TOF of the promoted catalysts. The 3,1,12 RuFeK achieved 16% conversion at 350 °C and 74% conversation at 400 °C. In contrast, the 1,3,12 RuFeK achieved 42% conversion at 400 °C. 

#### 3.3.3. Effects of K, Fe and Sr on the Adsorption of CO on Ru

**Figure 8:**
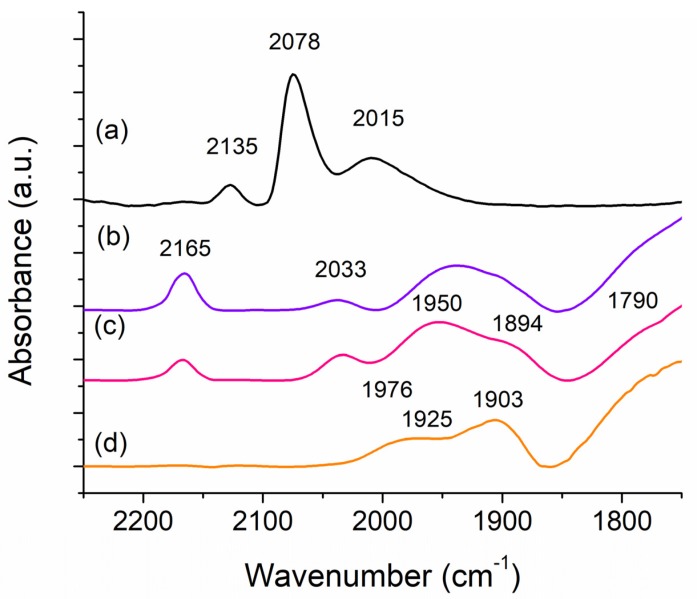
FT-IR spectra of CO adsorption on (**a**) 4 Ru (**b**) 4,12 RuK (**c**) 1,3,12 RuSrK and (**d**) 1,3,12 RuFeK.

The adsorption of CO as a probe molecule on these catalysts was investigated through FT-IR spectroscopy to further understand the differences between the Sr and Fe based catalysts. The spectra of room temperature CO adsorption for 1,3,12 RuSrK, 1,3,12 RuFeK are shown in Figure 8 in comparison to the 4 Ru and 4,12 RuK catalyst.

The 4,12 RuK catalyst that is shown in Figure 8b exhibited four primary features: a strong band at 2165 cm^−1^, a weak band at 2033 cm^−1^, a broad feature between 1995 cm^−1^ and 1850 cm^−1^, and a shoulder located at 1790 cm^−1^. The peak at 2033 cm^−1^ is commonly attributed to linearly adsorbed CO on metallic Ru and its peak position has been shown to be a function of CO coverage [79,80,81,82,83], due to the dipole-dipole interactions between neighboring adsorbed CO molecules [84]. This peak is also present in the 1,3,12 RuSrK spectra (Figure 8c), but is absent in the 1,3,12 RuFeK (Figure 8d). In the 1,3,12 RuFeK spectrum, the peak maximum at 1975 cm^−1^ may be attributed to the adsorption of CO on Fe [85]. Additionally, we see the suppression of any absorption bands in the 2200 to 2000 cm^−1^ region. Fe might act to suppress the adsorption of CO on Ru, as this has been reported to occur with the addition of Fe to Rh/SiO_2_ [86,87]. Interestingly, the relative intensity of the band at 2033 cm^−1^ is relatively larger in the Sr spectrum in comparison to the 4,12 RuK, even with a lowered number of adsorption sites, thus suggesting that the addition of Sr changes the concentration of sites that will linearly adsorb CO, and that the addition of Fe causes their suppression. 

Other studies of CO adsorption on Ru catalysts often report a weak band at about 2135 cm^−1^ in conjunction with a stronger band at 2078 cm^−1^, which are attributed to the CO vibration of multicarbonyl surface species [79,80,81]. This band is evident for the 4 Ru catalyst, but it is absent upon the addition of K to the catalysts, which has been previously reported [83,84]. The 4 Ru catalyst additionally contains a broad band at 2015 cm^−1^ that is suppressed with K addition, and it is related to the vibration of dicarbonyl species, as well as adsorbed CO on under-coordinated Ru, or high energy defect sites [81]. The suppression of these peaks with the addition of K has been hypothesized to be due to the blocking of under-coordinated Ru sites, which in turn suppresses hydrogen adsorption [84]. This is further evidenced by the reduction in H_2_ uptake that was exhibited in the 4,12 RuK catalyst compared to the 4 Ru catalyst (Table 1). 

Alkali addition to catalysts has also been known to show largely new interactions with CO in the low frequency range. This might be due to the interaction of CO with an electropositive center, which might weaken the CO bond and make it more reactive. Additives to metal catalysts have previously been shown to form new IR features due to the interaction between CO and the additive, at frequencies lower than CO adsorbed on the unpromoted metal [84]. The range of adsorption and bonding configuration of CO on K pre-covered Ru(0001) surface, for example, has been shown through high resolution electron energy loss spectroscopy (HREELS) to be a function of both the CO and K coverage and that K addition can shift the CO stretch frequencies from 2000 cm^−1^ to as low as 1400 cm^−1^ [88,89,90]. Other studies of CO adsorption of K-Ru catalysts have reported bands at 1995 cm^−1^, 1950 cm^−1^, and 1940 cm^−1^ [83,84], as well as the broadening of peaks and more asymmetrical spectra with the addition of K [83]. K addition weakens the CO bond and strengthens the Ru-CO bond. Therefore, the band at 2165 cm^−1^ in the 4,12 RuK and 1,3,12 RuSrK spectra might be due to the linearly adsorbed CO on partially oxidized Ru^n+^ (n = 1–3) which has been previously reported as such [79,81]. Here, the Ru might become partially oxidized Ru^n+^ from the adsorption and subsequent dissociation of CO due to the weakening of the CO bond with the addition of K.

With the further addition of Sr and Fe, we see changes in the relative intensities of the absorption bands in the 2000 cm^−1^ to 1850 cm^−1^ region, as shown in Figure 8c,d, respectively. The addition of Sr results in two distinct peak maxima located at 1950 cm^−1^ and 1894 cm^−1^, with respect to the 4,12 RuK catalyst. In contrast, the 1,3,12 RuFeK catalyst exhibits a shift to higher frequencies of this absorption region, with three peak maxima being located at 1976 cm^−1^, 1925 cm^−1^, and 1903 cm^−1^. Additionally, the shoulder located at 1790 cm^−1^ in 1,3,12 RuSrK and 4,12 RuK spectra can be attributed to bridge bonded CO [81,90]. This stretch is not observed for the 4 Ru catalyst (Figure 8a). This stretch has previously been reported to be broader and weaker than reported here [83,91], but these studies only use 3 wt% K, as well as different synthesis methods. Single crystal studies have shown a dramatic increase in bridge bonded CO on Pt(111) and Rh(111) as alkali coverage on these metals increases [92,93]. Therefore, the electropositive nature of Sr might act to similarly weaken the CO bond through further charge transfer, thus resulting in a more prominent low frequency band centered at 1880 cm^−1^, which results in a weakened CO bond, as well an increase in the amount bridge bonded CO with such low Ru content, as compared to 4,12 RuK catalyst.

## 4. Conclusions

We demonstrated the utilization of high throughput screening to determine low cost substitutional materials for low temperature ammonia decomposition. This screen concluded that substitution of Ru with Mg, Ca, Sr, Sc, Y, Ta, Hf, and Zr resulted in catalyst formulations that were more active than a baseline 4,12 RuK catalyst with less Ru content. Additionally, we were able to determine that, with the addition of Y, Hf, Zr, and Sr, the Ru content could be lowered by a factor of four, without an apparent loss in activity. XRD analysis confirmed that these catalysts contained Ru mixed metal oxides of the form KRu_4_O_8_ and KRuO_4_. These structures enable close contact between the K promoter and the Ru active sites, thus enhancing electron donation from K to Ru and modifying the reactivity of the catalysts.

Further investigation into the Sr and Fe based catalysts confirmed that the addition of Sr did not act to change the apparent activation energy of the catalyst with each of the three weight loadings studied. Additionally, FT-IR analysis of CO adsorption revealed that the addition of Sr resulted in weakened CO bonds on Ru sites due to its electropositive nature, being accompanied by strongly adsorbed linear CO. The addition of other electropositive elements, such as K, Li, and Na, has shown a positive effect on ammonia decomposition, and to further facilitate the RDS [94]. This might be analogous to the weakening of N-H bonds of adsorbed ammonia and thus facilitating the dehydrogenation of ammonia. A catalyst containing 1,3,12 RuSrK was shown to achieve a TOF of 1.78 s^−1^ at 40 °C, which is five times the TOF of 4,12 RuK at the same temperature. These materials were discovered through the systematic utilization of high throughput screening, which successfully resulted in catalysts that were highly active in 100% NH_3,_ which shows their potential for on-site H_2_ generation technologies.

## 5. Patents

Lauterbach J., McCullough K., “Ammonia Decomposition Catalyst Systems,” US Patent App.: 16/376,158; 2020.

## Figures and Tables

**Table 1:** H_2_ uptake, turnover frequencies (TOF) and apparent activation energies (E_a_) for Sr and Fe substituted catalysts as compared to 4,12 RuK and unpromoted 4 Ru catalyst.

Catalyst	H_2_ Uptake (μmol H_2_/g)	TOF (s^−1^)			E_a_ (kJ/mol)
		300 °C	350 °C	400 °C	
4 Ru	6.50	0.00	0.00	0.14	125.2 ± 8.9
4,12 RuK	4.70	0.13	0.26	0.33	65.7 ± 7.7
3,1,12 RuSrK	1.90	0.16	0.43	0.88	149.6 ± 4.1
2,2,12 RuSrK	0.90	0.38	0.81	1.38	153.7 ± 2.4
1,3,12 RuSrK	0.75	0.47	1.10	1.78	156.4 ± 1.6
3,1,12 RuFeK	0.76	0.00	0.35	0.97	248.1 ± 3.0
2,2,12 RuFeK	0.10	0.00	0.03	0.41	226.6 ± 2.9
1,3,12 RuFeK	1.20	0.00	0.00	0.28	250.9 ± 7.6

## Supplementary Materials

The following are available online at https://www.mdpi.com/1996-1944/13/8/1869/s1, Figure S1. Thermodynamic limit of ammonia decomposition at 101.325 kPa, Figure S2. Catalytic activity at 250 °C, Figure S3. Catalytic activity at 350 °C, Figure S4. Catalytic activity at 400 °C, Figure S5. Arrhenius plot for 4 Ru and 4,12 RuK, Table S1. Summary of the Ru species present in each catalyst, Figure S6. XRD patterns of (from bottom to top): 1,3,12 RuCuK, 2,2,12 RuCuK and 3,1,12 RuCuK, Figure S7. XRD patterns of (from bottom to top): 1,3,12 RuYK, 2,2,12 RuYK and 3,1,12 RuYK, Figure S8. XRD patterns of (from bottom to top): 1,3,12 RuAuK, 2,2,12 RuAuK and 3,1,12 RuAuK, Figure S9. XRD patterns of (from bottom to top): 1,3,12 RuNiK, 2,2,12 RuNiK and 3,1,12 RuNiK, Figure S10. XRD patterns of (from bottom to top): 1,3,12 RuInK, 2,2,12 RuInK and 3,1,12 RuInK, Figure S11. XRD patterns of (from bottom to top): 1,3,12 RuNbK, 2,2,12 RuNbK and 3,1,12 RuNbK, Figure S12. XRD patterns of (from bottom to top): 1,3,12 RuMnK, 2,2,12 RuMnK and 3,1,12 RuMnK, Figure S13. XRD patterns of (from bottom to top): 1,3,12 RuAgK, 2,2,12 RuAgK and 3,1,12 RuAgK, Figure S14. XRD patterns of (from bottom to top): 1,3,12 RuPtK, 2,2,12 RuPtK and 3,1,12 RuPtK, Figure S15. XRD patterns of (from bottom to top): 1,3,12 RuZrK, 2,2,12 RuZrK and 3,1,12 RuZrK, Figure S16. XRD patterns of (from bottom to top): 1,3,12 RuReK, 2,2,12 RuReK and 3,1,12 RuReK, Figure S17. XRD patterns of (from bottom to top): 1,3,12 RuIrK, 2,2,12 RuIrK and 3,1,12 RuIrK, Figure S18. XRD patterns of (from bottom to top): 1,3,12 RuCoK, 2,2,12 RuCoK and 3,1,12 RuCoK, Figure S19. XRD patterns of (from bottom to top): 1,3,12 RuWK, 2,2,12 RuWK and 3,1,12 RuWK, Figure S20. XRD patterns of (from bottom to top): 1,3,12 RuCaK, 2,2,12 RuCaK and 3,1,12 RuCaK, Figure S21. XRD patterns of (from bottom to top): 1,3,12 RuHfK, 2,2,12 RuHfK and 3,1,12 RuHfK, Figure S22. XRD patterns of (from bottom to top): 1,3,12 RuSnK, 2,2,12 RuSnK and 3,1,12 RuSnK, Figure S23. XRD patterns of (from bottom to top): 1,3,12 RuZnK, 2,2,12 RuZnK and 3,1,12 RuZnK, Figure S24. XRD patterns of (from bottom to top): 1,3,12 RuOsK, 2,2,12 RuOsK and 3,1,12 RuOsK, Figure S25. XRD patterns of (from bottom to top): 1,3,12 RuPbK, 2,2,12 RuPbK and 3,1,12 RuPbK, Figure S26. XRD patterns of (from bottom to top): 1,3,12 RuMoK, 2,2,12 RuMoK and 3,1,12 RuMoK, Figure S27. XRD patterns of (from bottom to top): 1,3,12 RuCrK, 2,2,12 RuCrK and 3,1,12 RuCrK, Figure S28. XRD patterns of (from bottom to top): 1,3,12 RuScK, 2,2,12 RuScK and 3,1,12 RuScK, Figure S29. XRD patterns of (from bottom to top): 1,3,12 RuPdK, 2,2,12 RuPdK and 3,1,12 RuPdK, Figure S30. XRD patterns of (from bottom to top): 1,3,12 RuMgK, 2,2,12 RuMgK and 3,1,12 RuMgK, Figure S31. XRD patterns of (from bottom to top): 1,3,12 RuBiK, 2,2,12 RuBiK and 3,1,12 RuBiK, Figure S32. XRD patterns of (from bottom to top): 1,3,12 RuCdK, 2,2,12 RuCdK and 3,1,12 RuCdK.
