# Supplementary material for: Material Discovery and High Throughput Exploration of Ru Based Catalysts for Low Temperature Ammonia Decomposition
Source: Materials (Basel). 2020 Apr 16;13(8):1869. doi: 10.3390/ma13081869 (PMC7215519; doi:10.3390/ma13081869)
Supplement: Supplementary file 1 [file materials-13-01869-s001.pdf]

# Supplemental Information

*Article*

# Material Discovery and High Throughput Exploration of Ru Based Catalysts for Low Temperature Ammonia Decomposition

**Katherine McCullough, Pei-Hua Chiang, Juan D. Jimenez, and Jochen A. Lauterbach ***

Department of Chemical Engineering University of South Carolina, Columbia, SC 29208, USA; mccullke@email.sc.edu (K.M.); pchiang@email.sc.edu (P.C.); jiminezj@email.sc.edu (J.D.J.)

* Correspondence: lauteraj@cec.sc.edu

Received: 21 February 2020; Accepted: 14 April 2020; Published: date

Thermodynamic Limit

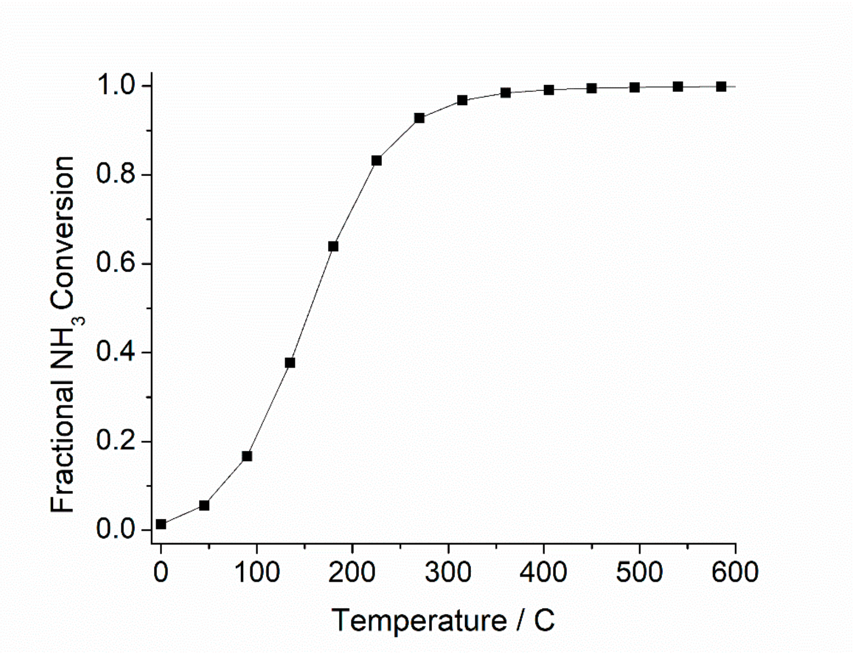


Figure S1. Thermodynamic limit of ammonia decomposition at 101.325 kPa total pressure.

High throughput screening results

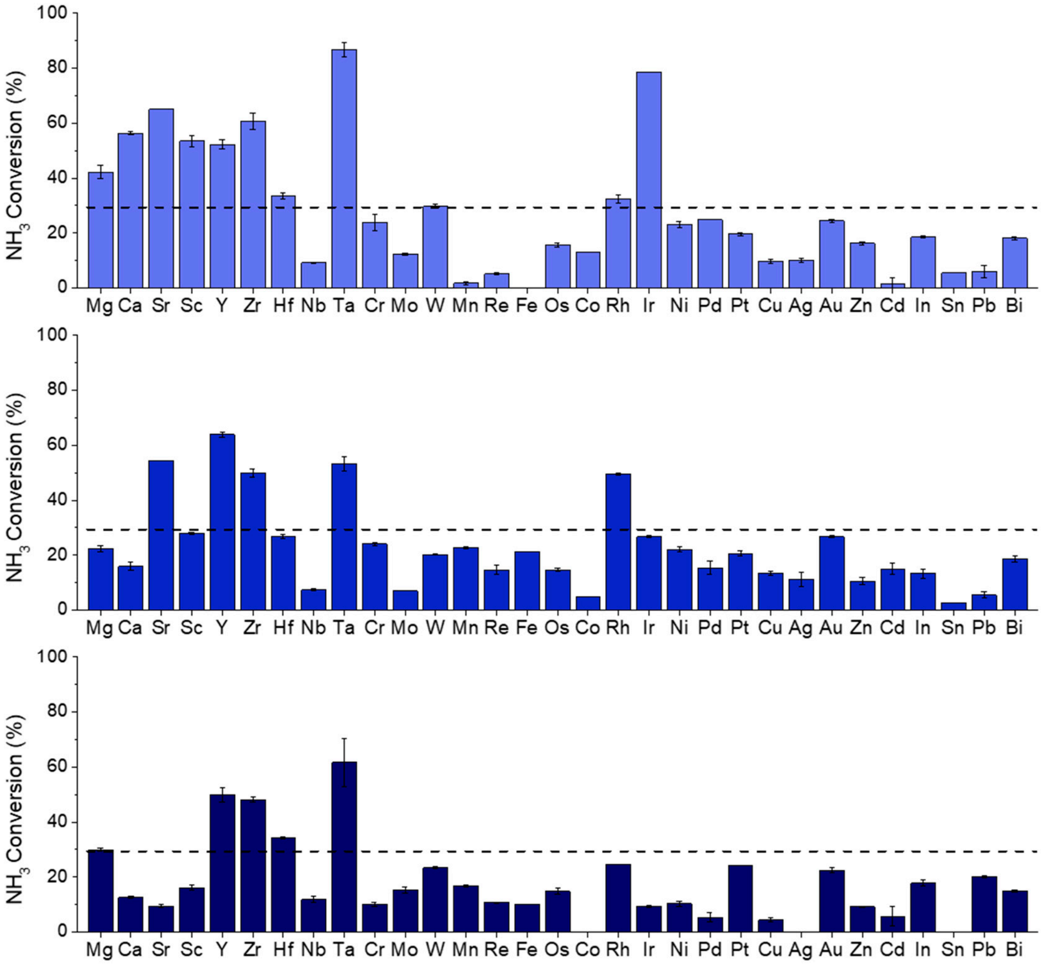


Figure S2. Catalytic activity at 250ºC. From bottom to top: 3,1,12 RuMK, 2,2,12 RuMK, and 1,3,12 RuMK, where M is the substituted metal, denoted in the x axis. The black dashed line indicates the activity of the baseline 4,12 RuK catalyst at 250ºC. Reaction conditions: 1% NH3 in balance Ar, 30,000 mL/hr/gcat, 1 bar.

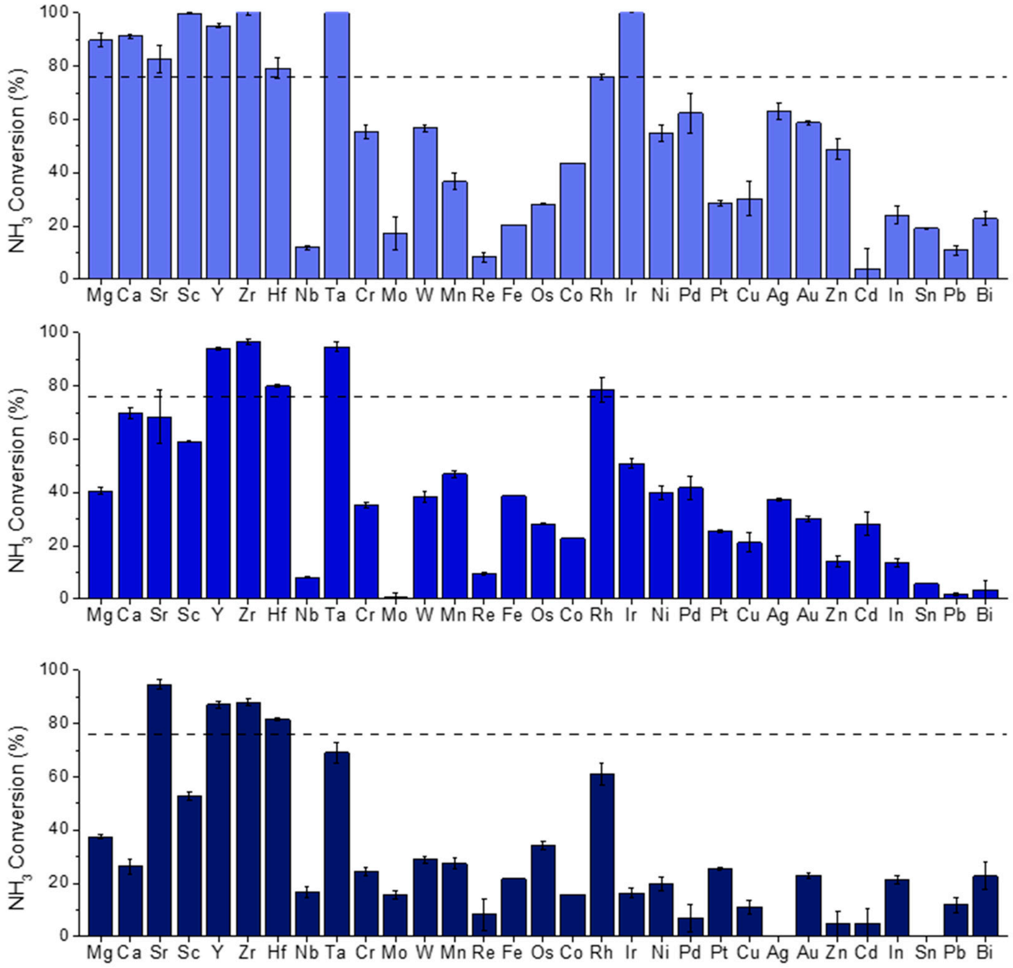


Figure S3. Catalytic activity at 350°C. From bottom to top: 3,1,12 RuMK, 2,2,12 RuMK, and 1,3,12 RuMK, where M is the substituted metal, denoted in the x axis. The black dashed line indicates the activity of the baseline 4,12 RuK catalyst at 350°C. Reaction conditions: 1% NH3 in balance Ar, 30,000 mL/hr/gcat, 1 bar.

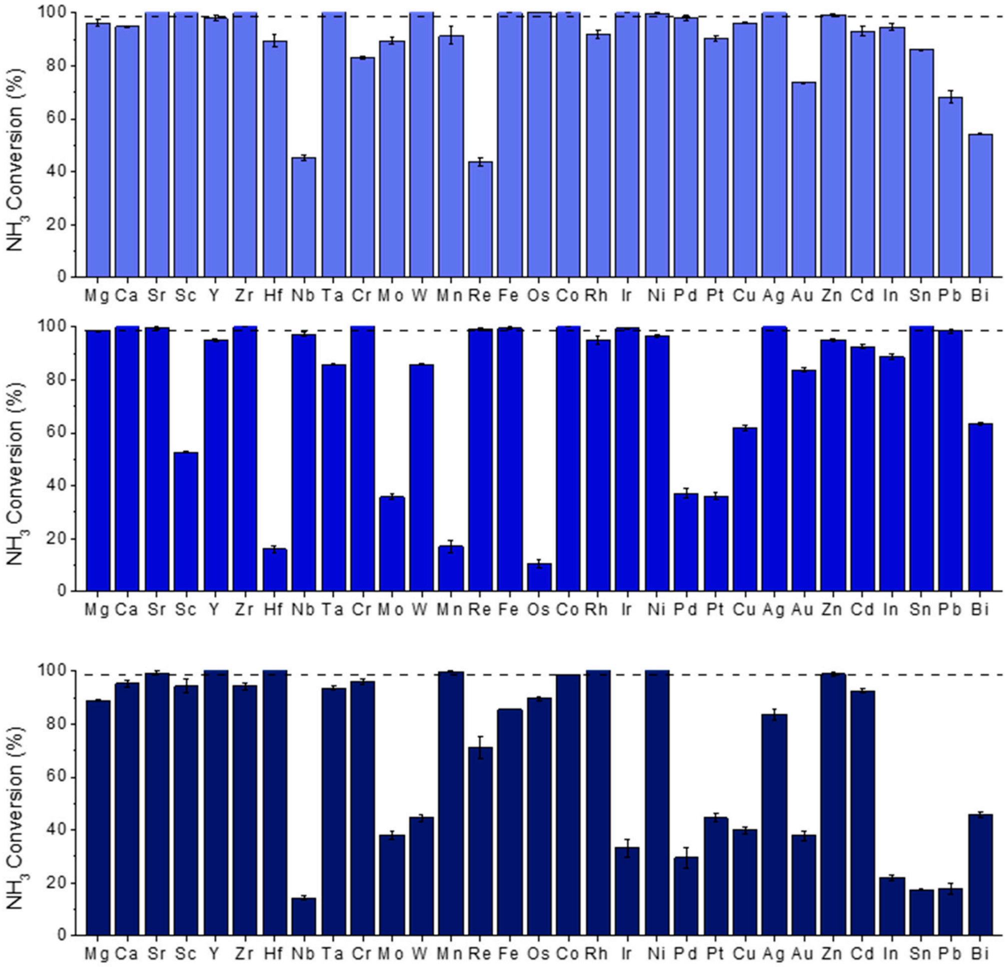


Figure S4. Catalytic activity at 400ºC. From bottom to top: 3,1,12 RuMK, 2,2,12 RuMK, and 1,3,12 RuMK, where M is the substituted metal, denoted in the x axis. The black dashed line indicates the activity of the baseline 4,12 RuK catalyst at 400ºC. Reaction conditions: 1% NH3 in balance Ar, 30,000 mL/hr/gcat, 1 bar.

Evaluation of Apparent Activation Energy

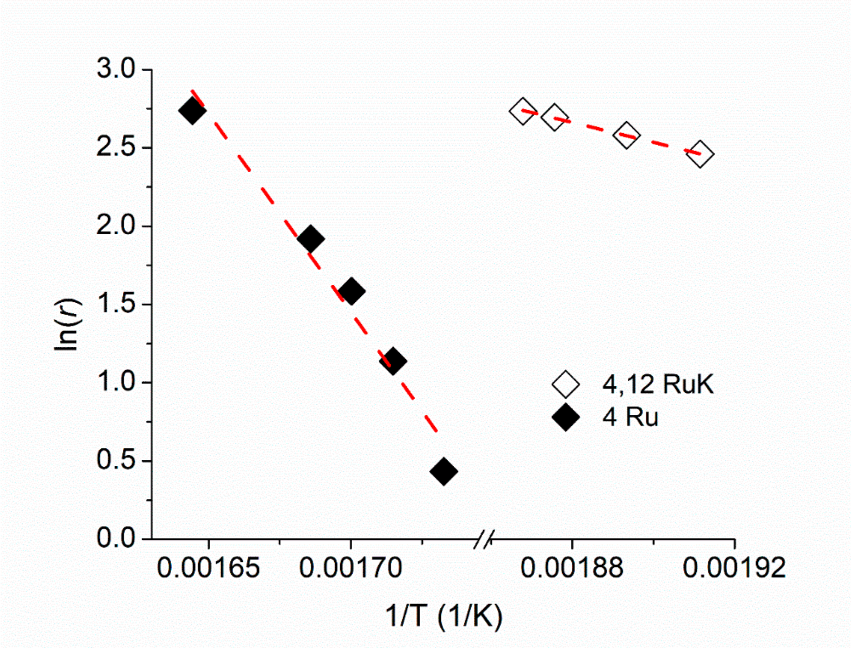


Figure S5. Arrhenius plot for 4 Ru (filled diamonds) and 4,12 RuK (open diamonds). Reaction conditions: 100% $NH_3$, 5,400 mL/hr/$g_{cat}$, and 1 bar. Measurements conducted under differential conditions.

Internal Mass Transfer Resistance

The Weis–Prater criterion was used to determine the absence of internal diffusion resistances. The Weis–Prater criterion is given by:

$$\frac{r'_A \rho_c R_P^2}{D_e C_{AS}} \ll 1$$

where $r'_A$= measured $NH_3$ reaction rate (mol/kg/s), $\rho_c$= catalyst pellet density (750 kg/m$^3$), $R_P^2$= pellet radius (2.25x10$^{-5}$ m), $D_e$=effective diffusivity of $NH_3$ in the catalyst pellet, and $C_{AS}$= $NH_3$ concentration at the pellet surface (16.7 mol/m$^3$). Effective diffusivity is calculated by:

$$D_e = \frac{D_{NH3-H2}\phi_p \sigma}{\tau}$$

where $\phi_p$= pellet porosity, $\sigma$ is the constriction factor, and $\tau$ is the tortuosity. Typical values for a catalyst pellet are $\phi_p$=0.4, $\sigma$=0.8, and $\tau$= 3 (values adapted from ref. 65). $D_{NH3-H2}$ is the diffusion coefficient of $NH_3$ in $H_2$ at 400°C and it is given by:

$$D_{NH3-H2} = \frac{0.00266T^{3/2}}{PM_{NH3-H2}^{1/2}\sigma_{NH3-H2}^{2}\Omega_D}$$

where T is the temperature (K), P is the pressure (bar), $M_{NH3-H2}$=2*[(1/$M_{NH3}$)+(1/$M_{H2}$)]$^{-1}$, $M_{NH3}$=molecular weight of $NH_3$, $M_{H2}$ = molecular weight of $H_2$, $\sigma_{NH3-H2}$ = characteristic length (Å), and $\Omega_D$ is the diffusion collision integral (dimensionless) (adapted from ref. 74). $D_e$ was found to be 3.51 x$10^{-4}$ $m^2$/s.

The measured reaction rate at 400°C for the unpromoted 4 Ru catalyst is 2.07 mol/kg/s. The LHS of the Weis–Prater criterion equates to 1.34x$10^{-3}$, which is much less than 1. Therefore, the criterion is satisfied.

XRD Analysis

Refer to main text for references for patterns of $RuO_2$, $KRuO_4$, KCl, and $KRu_4O_8$. Fig. S6–S32 show the XRD patterns for each catalyst. The CIF file no. is given for other species that are indexed in a pattern. . Table S1 is given to summarize the Ru species present in each catalyst, and additional speciation can be found with each XRD pattern.  In instances where there are no entries in the table for a certain metal, there were no detectable Ru species present in the XRD patterns.

Table S1. Summary of the Ru species present in each catalyst

| Ru, Secondary Metal,K Weight Loading | Secondary Metal | $RuO_2$ | $KRuO_4$ | $KRu_4O_8$ |
|---|---|---|---|---|
| 3,1,12 | **Mg** | Y | | Y |
| 2,2,12 | | Y | | |
| 1,3,12 | | Y | | |
| 3,1,12 | **Ca** | | | |
| 2,2,12 | | | | |
| 1,3,12 | | | Y | |
| 3,1,12 | **Sr** | | Y | |
| 2,2,12 | | | Y | |
| 1,3,12 | | | | |
| 3,1,12 | **Sc** | Y | Y | |
| 2,2,12 | | Y | | |
| 1,3,12 | | Y | | |
| 3,1,12 | **Y** | | Y | |
| 2,2,12 | | | Y | |
| 1,3,12 | | | Y | |
| 3,1,12 | **Zr** | Y | | |
| 2,2,12 | | Y | Y | |
| 1,3,12 | | Y | Y | |
| 3,1,12 | **Hf** | | | |
| 2,2,12 | | | Y | |
| 1,3,12 | | | Y | |
| 3,1,12 | **Nb** | Y | | |
| 2,2,12 | | Y | | |
| 1,3,12 | | Y | | |
| 3,1,12 | **Cr** | Y | | Y |
| 2,2,12 | | Y | | |
| 1,3,12 | | | | |
| 3,1,12 | **Mo** | Y | Y | |
| 2,2,12 | | Y | | |
| 1,3,12 | | Y | | |
| 3,1,12 | **W** | Y | | |
| 2,2,12 | | Y | | |
| 1,3,12 | | Y | | |

| | | | | |
|---|---|---|---|---|
| 3,1,12 | **Mn** | | | Y |
| 2,2,12 | | | | Y |
| 1,3,12 | | | | Y |
| 3,1,12 | **Re** | | | |
| 2,2,12 | | Y | | |
| 1,3,12 | | Y | | |
| 3,1,12 | **Fe** | | | Y |
| 2,2,12 | | | | Y |
| 1,3,12 | | | | |
| 3,1,12 | **Os** | | | Y |
| 2,2,12 | | | | Y |
| 1,3,12 | | | | |
| 3,1,12 | **Co** | | | Y |
| 2,2,12 | | Y | | |
| 1,3,12 | | | | |
| 3,1,12 | **Ir** | | | Y |
| 2,2,12 | | | Y | Y |
| 1,3,12 | | | Y | Y |
| 3,1,12 | **Ni** | Y | | |
| 2,2,12 | | Y | | |
| 1,3,12 | | | | |
| 3,1,12 | **Pd** | Y | | Y |
| 2,2,12 | | Y | | Y |
| 1,3,12 | | Y | | Y |
| 3,1,12 | **Pt** | Y | | |
| 2,2,12 | | Y | | |
| 1,3,12 | | Y | | |
| 3,1,12 | **Cu** | Y | | |
| 2,2,12 | | Y | | |
| 1,3,12 | | | | |
| 3,1,12 | **Ag** | | | |
| 2,2,12 | | | | |
| 1,3,12 | | | | |
| 3,1,12 | **Au** | Y | | |
| 2,2,12 | | Y | | |
| 1,3,12 | | Y | | |
| 3,1,12 | **Zn** | | Y | |
| 2,2,12 | | Y | | |
| 1,3,12 | | Y | | |
| 3,1,12 | **Cd** | | Y | |
| 2,2,12 | | | Y | |
| 1,3,12 | | | Y | |
| 3,1,12 | **In** | Y | | |
| 2,2,12 | | Y | | |
| 1,3,12 | | Y | | |
| 3,1,12 | **Sn** | | | |

| | | | | |
|---|---|---|---|---|
| 2,2,12 | | Y | | |
| 1,3,12 | | | | |
| 3,1,12 | **Pb** | | | |
| 2,2,12 | | | | |
| 1,3,12 | | | | |
| 3,1,12 | **Bi** | | | |
| 2,2,12 | | | | |
| 1,3,12 | | | | |

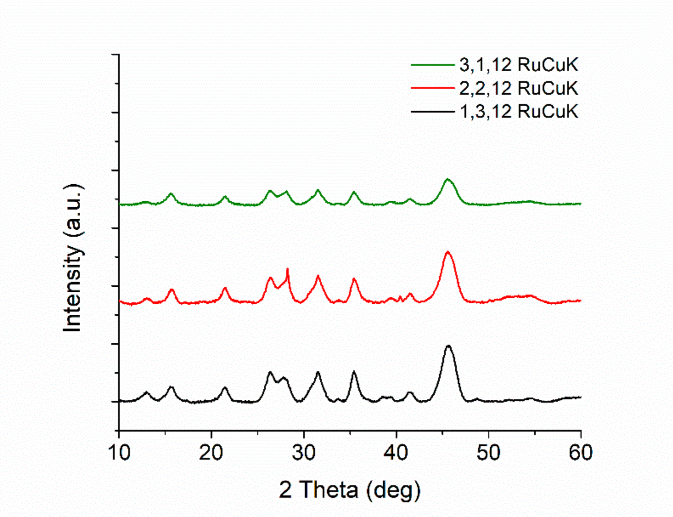


Figure S6. XRD patterns of (from bottom to top): 1,3,12 RuCuK, 2,2,12 RuCuK, and 3,1,12 RuCuK

| Phase | cif no. | Present in: | | |
|---|---|---|---|---|
| | | 3,1,12 | 2,2,12 | 1,3,12 |
| $RuO_2$ | | Y | Y | |
| $KRu_4O_8$ | | | | |
| $KRuO_4$ | | | | |
| KCl | | | | |
| $KCuO_2$ | 96-153-0994 | Y | Y | Y |
| $KClO_2$ | 96-201-4619 | Y | Y | Y |

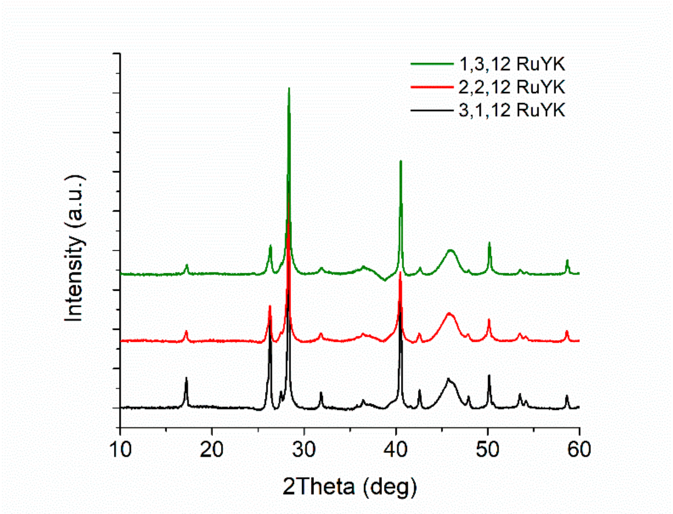


Figure S7. XRD patterns of (from bottom to top): 1,3,12 RuYK, 2,2,12 RuYK, and 3,1,12 RuYK

| Phase | cif no. | Present in: | | |
|---|---|---|---|---|
| | | 3,1,12 | 2,2,12 | 1,3,12 |

| | | | | |
|---|---|---|---|---|
| $RuO_2$ | | | | |
| $KRu_4O_8$ | | | | |
| $KRuO_4$ | | Y | Y | Y |
| KCl | | Y | Y | Y |

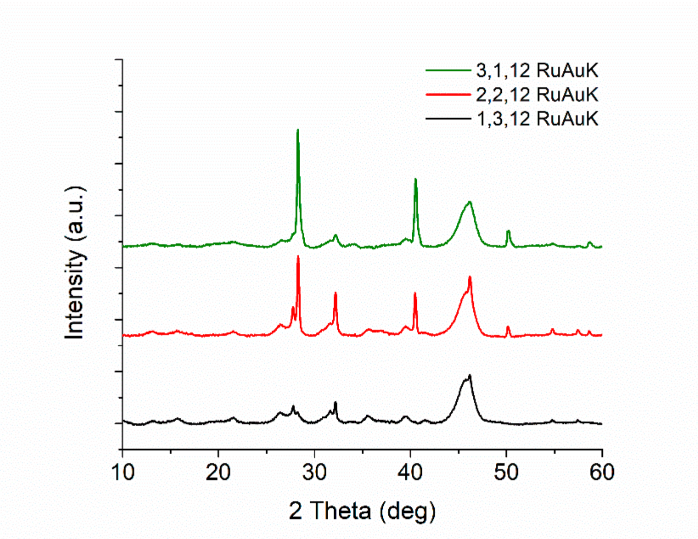


Figure S8. XRD patterns of (from bottom to top): 1,3,12 RuAuK, 2,2,12 RuAuK, and 3,1,12 RuAuK

| Phase | cif no. | Present in: | | |
|---|---|---|---|---|
| | | 3,1,12 | 2,2,12 | 1,3,12 |
| $RuO_2$ | | Y | Y | Y |
| $KRu_4O_8$ | | | | |
| $KRuO_4$ | | | | |
| KCl | | Y | Y | Y |
| Au | 96-900-8464 | Y | Y | Y |

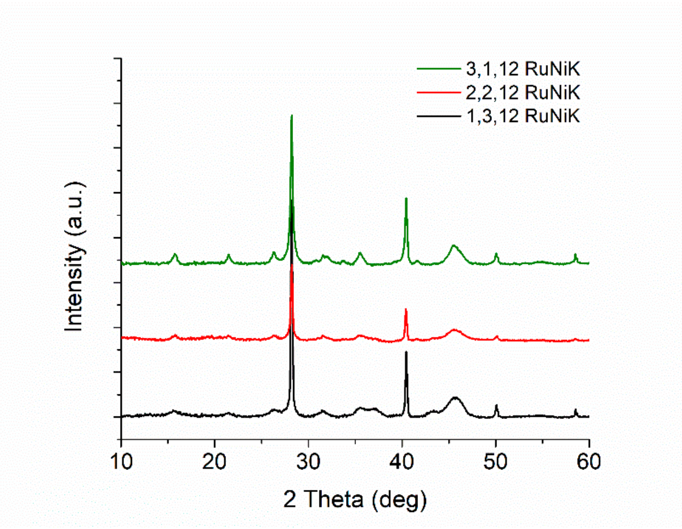


Figure S9. XRD patterns of (from bottom to top): 1,3,12 RuNiK, 2,2,12 RuNiK, and 3,1,12 RuNiK

| Phase | cif no. | Present in: | | |
|---|---|---|---|---|
| | | 3,1,12 | 2,2,12 | 1,3,12 |
| $RuO_2$ | | Y | Y | |
| $KRu_4O_8$ | | | | |
| $KRuO_4$ | | | | |
| KCl | | Y | Y | Y |

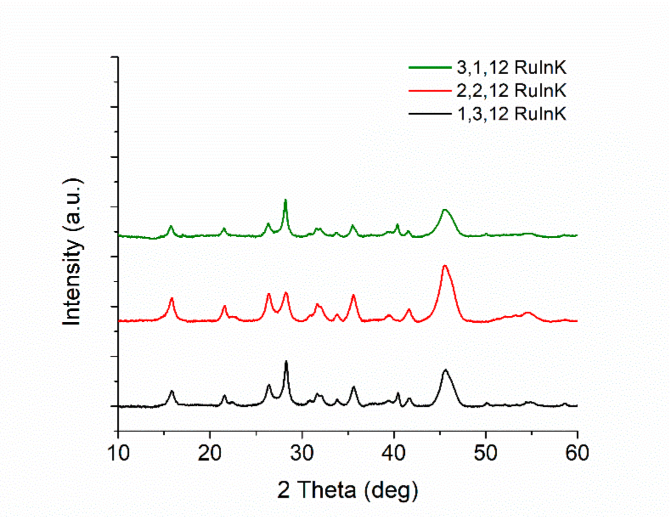


Figure S10. XRD patterns of (from bottom to top): 1,3,12 RuInK, 2,2,12 RuInK, and 3,1,12 RuInK

| Phase | cif no. | Present in: | | |
|---|---|---|---|---|
| | | 3,1,12 | 2,2,12 | 1,3,12 |
| $RuO_2$ | | Y | Y | Y |
| $KRu_4O_8$ | | | | |
| $KRuO_4$ | | | | |
| KCl | | Y | | Y |
| $In_2O_3$ | 96-900-3113 | | Y | Y |

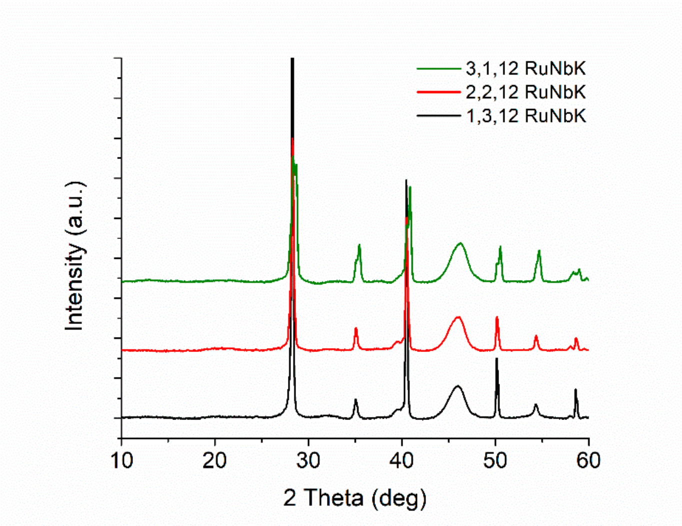


Figure S11. XRD patterns of (from bottom to top): 1,3,12 RuNbK, 2,2,12 RuNbK, and 3,1,12 RuNbK

| Phase | cif no. | Present in: | | |
|---|---|---|---|---|
| | | 3,1,12 | 2,2,12 | 1,3,12 |
| $RuO_2$ | | Y | Y | Y |
| $KRu_4O_8$ | | | | |
| $KRuO_4$ | | | | |
| KCl | | Y | Y | Y |

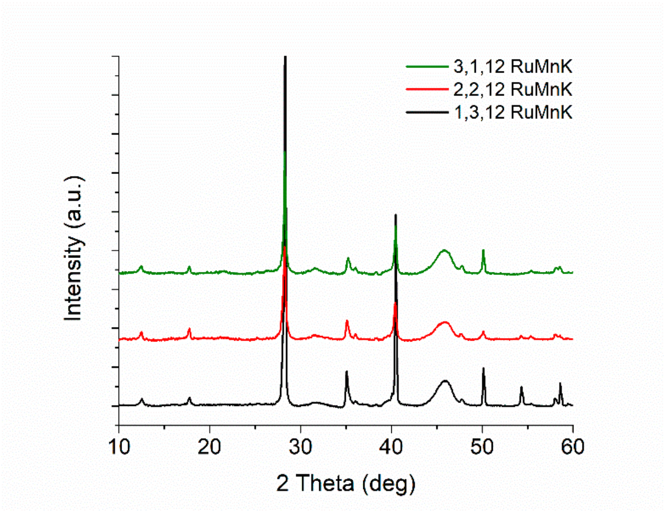


Figure S12. XRD patterns of (from bottom to top): 1,3,12 RuMnK, 2,2,12 RuMnK, and 3,1,12 RuMnK

| Phase | cif no. | Present in: | | |
|---|---|---|---|---|
| | | 3,1,12 | 2,2,12 | 1,3,12 |
| $RuO_2$ | | | | |
| $KRu_4O_8$ | | Y | Y | Y |
| $KRuO_4$ | | | | |
| KCl | | Y | Y | Y |

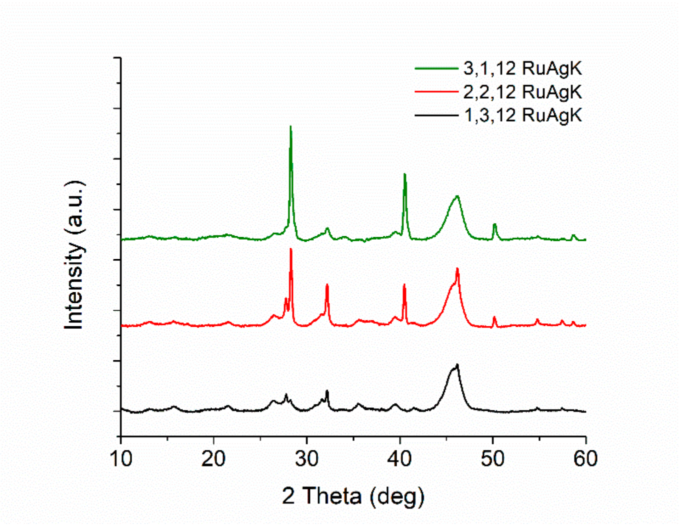


Figure S13. XRD patterns of (from bottom to top): 1,3,12 RuAgK, 2,2,12 RuAgK, and 3,1,12 RuAgK

| Phase | cif no. | Present in: | | |
|---|---|---|---|---|
| | | 3,1,12 | 2,2,12 | 1,3,12 |
| $RuO_2$ | | | | |
| $KRu_4O_8$ | | | | |
| $KRuO_4$ | | | | |
| KCl | | Y | Y | Y |
| AgCl | 96-901-1667 | Y | Y | Y |

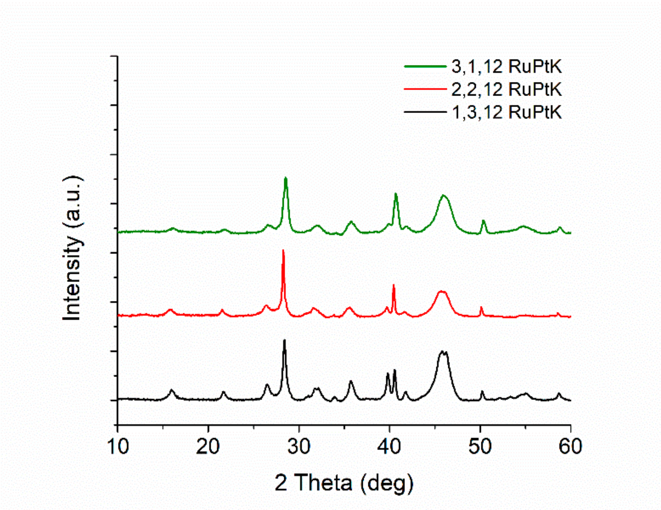


Figure S14. XRD patterns of (from bottom to top): 1,3,12 RuPtK, 2,2,12 RuPtK, and 3,1,12 RuPtK

| Phase | cif no. | Present in: | | |
|---|---|---|---|---|
| | | 3,1,12 | 2,2,12 | 1,3,12 |
| $RuO_2$ | | Y | Y | y |
| $KRu_4O_8$ | | | | |
| $KRuO_4$ | | | | |
| KCl | | Y | Y | Y |
| Pt | 96-900-8481 | Y | Y | Y |

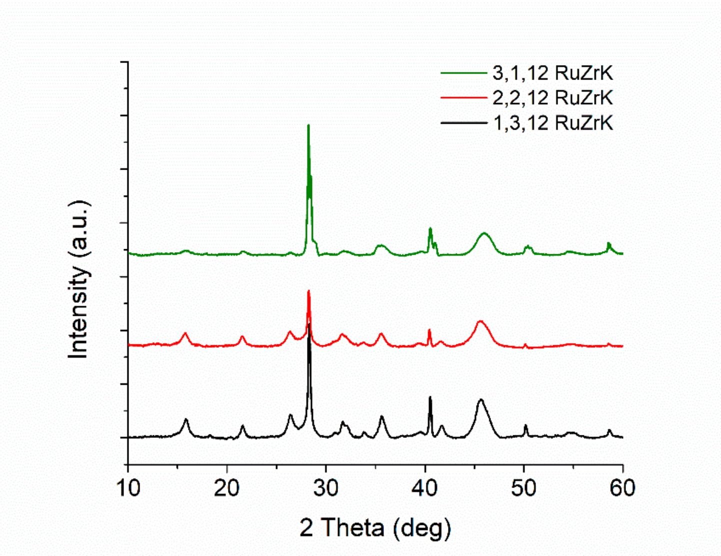


Figure S15. XRD patterns of (from bottom to top): 1,3,12 RuZrK, 2,2,12 RuZrK, and 3,1,12 RuZrK

| Phase | cif no. | Present in: | | |
|---|---|---|---|---|
| | | 3,1,12 | 2,2,12 | 1,3,12 |
| $RuO_2$ | | Y | Y | Y |
| $KRu_4O_8$ | | | | |
| $KRuO_4$ | | | Y | Y |
| KCl | | Y | Y | Y |
| $ZrO_2$ | 96-900-5834 | Y | Y | Y |

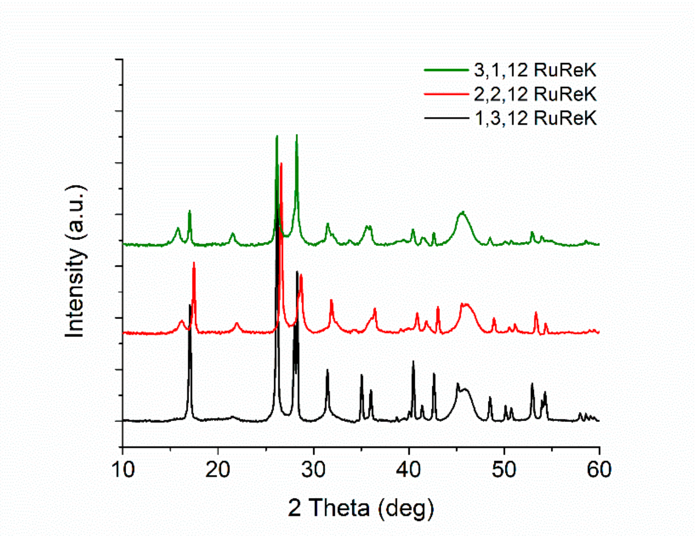


Figure S16. XRD patterns of (from bottom to top): 1,3,12 RuReK, 2,2,12 RuReK, and 3,1,12 RuReK

| Phase | cif no. | Present in: | | |
|---|---|---|---|---|
| | | 3,1,12 | 2,2,12 | 1,3,12 |
| $RuO_2$ | | | Y | Y |
| $KRu_4O_8$ | | | | |
| $KRuO_4$ | | | | |
| KCl | | Y | Y | Y |
| $KReO_4$ | 96-200-1935 | Y | Y | Y |

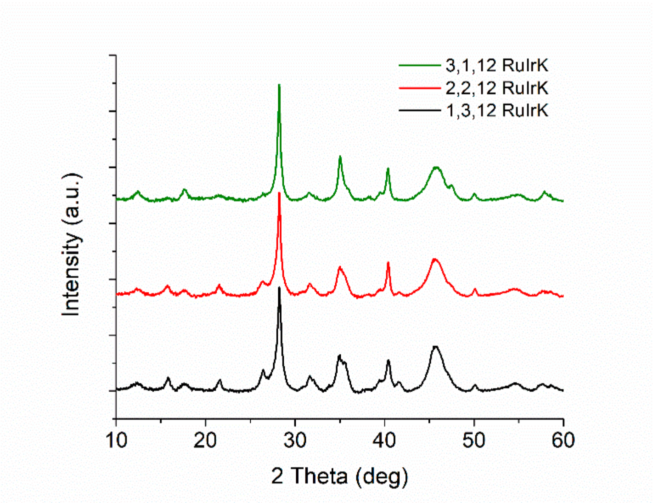


Figure S17. XRD patterns of (from bottom to top): 1,3,12 RuIrK, 2,2,12 RuIrK, and 3,1,12 RuIrK

| Phase | cif no. | Present in: | | |
|---|---|---|---|---|
| | | 3,1,12 | 2,2,12 | 1,3,12 |
| $RuO_2$ | | | | |
| $KRu_4O_8$ | | Y | Y | Y |
| $KRuO_4$ | | | Y | Y |
| KCl | | Y | Y | Y |
| $KIr_4O_8$ | 96-433-8123 | Y | Y | Y |

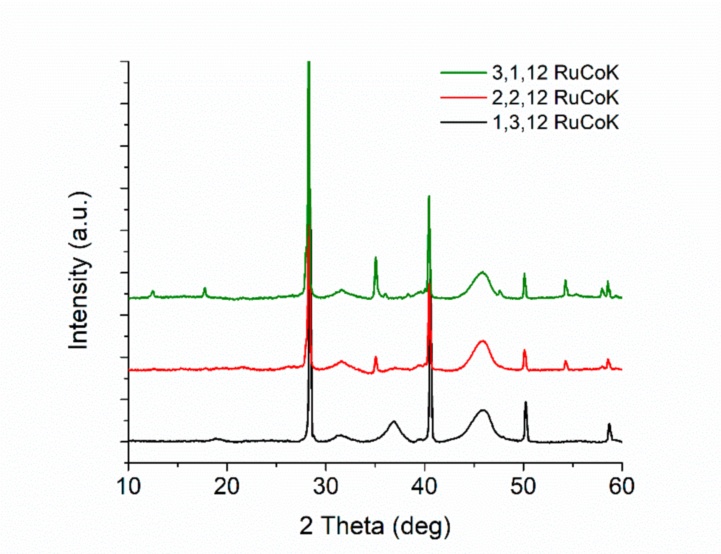


Figure S18. XRD patterns of (from bottom to top): 1,3,12 RuCoK, 2,2,12 RuCoK, and 3,1,12 RuCoK

| Phase | cif no. | Present in: | | |
|---|---|---|---|---|
| | | 3,1,12 | 2,2,12 | 1,3,12 |
| $RuO_2$ | | | Y | |
| $KRu_4O_8$ | | Y | | |
| $KRuO_4$ | | | | |
| KCl | | Y | Y | Y |
| $Co_3O_4$ | 96-153-4892 | | Y | Y |

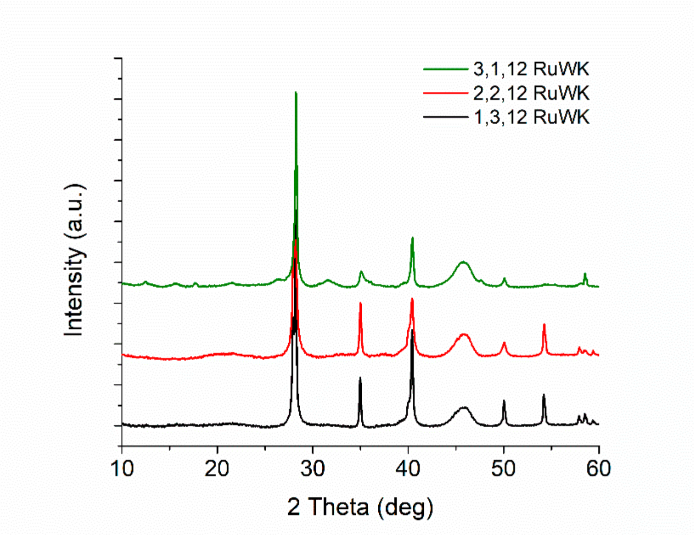


Figure S19. XRD patterns of (from bottom to top): 1,3,12 RuWK, 2,2,12 RuWK, and 3,1,12 RuWK

| Phase | cif no. | Present in: | | |
|---|---|---|---|---|
| | | 3,1,12 | 2,2,12 | 1,3,12 |
| $RuO_2$ | | Y | Y | Y |
| $KRu_4O_8$ | | | | |
| $KRuO_4$ | | | | |
| KCl | | Y | Y | Y |

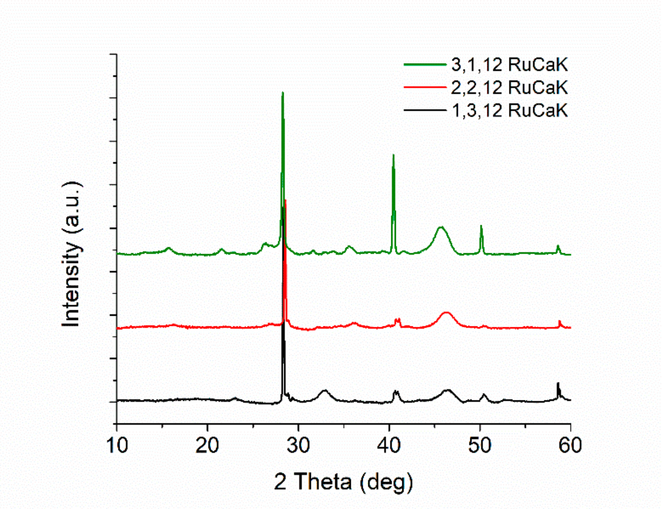


Figure S20. XRD patterns of (from bottom to top): 1,3,12 RuCaK, 2,2,12 RuCaK, and 3,1,12 RuCaK

| Phase | cif no. | Present in: | | |
|---|---|---|---|---|
| | | 3,1,12 | 2,2,12 | 1,3,12 |
| $RuO_2$ | | | | |
| $KRu_4O_8$ | | | | |
| $KRuO_4$ | | Y | | |
| KCl | | Y | Y | Y |

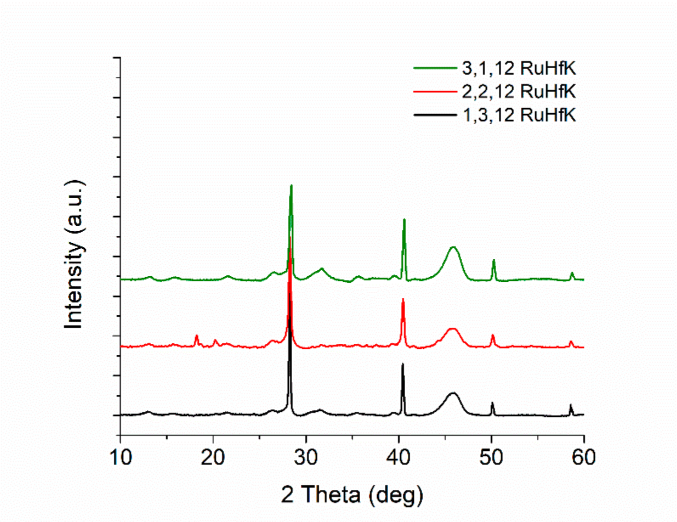


Figure S21. XRD patterns of (from bottom to top): 1,3,12 RuHfK, 2,2,12 RuHfK, and 3,1,12 RuHfK

| Phase | cif no. | Present in: | | |
|---|---|---|---|---|
| | | 3,1,12 | 2,2,12 | 1,3,12 |
| $RuO_2$ | | | | |
| $KRu_4O_8$ | | | | |
| $KRuO_4$ | | Y | Y | |
| KCl | | Y | Y | Y |

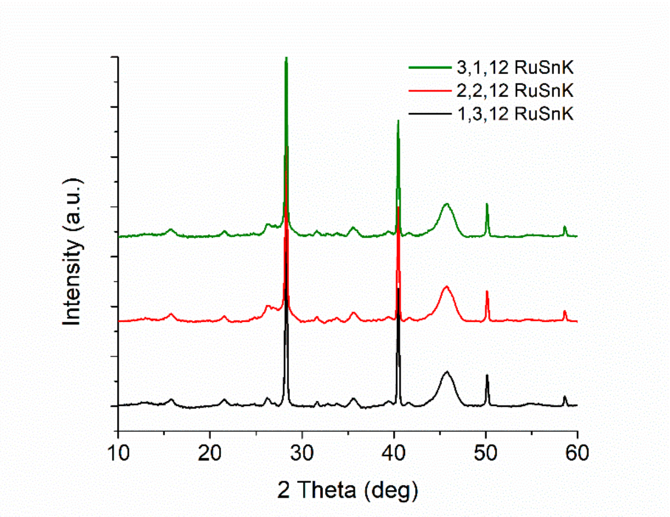


Figure S22. XRD patterns of (from bottom to top): 1,3,12 RuSnK, 2,2,12 RuSnK, and 3,1,12 RuSnK

| Phase | cif no. | Present in: | | |
|---|---|---|---|---|
| | | 3,1,12 | 2,2,12 | 1,3,12 |
| $RuO_2$ | | | Y | |
| $KRu_4O_8$ | | | | |
| $KRuO_4$ | | | | |
| KCl | | Y | Y | Y |

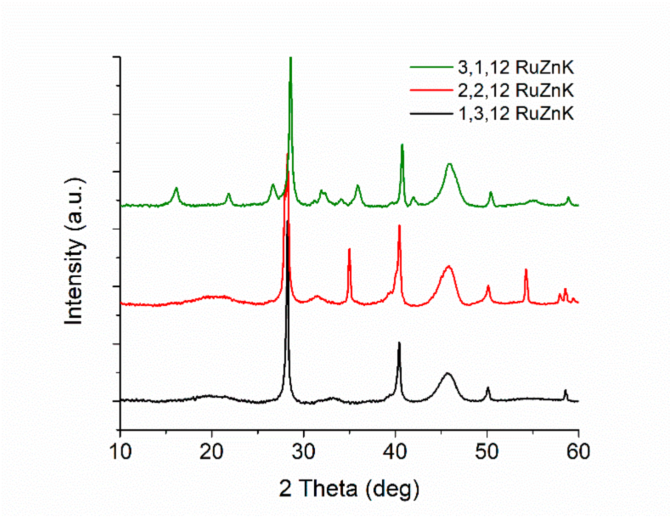


Figure S23. XRD patterns of (from bottom to top): 1,3,12 RuZnK, 2,2,12 RuZnK, and 3,1,12 RuZnK

| Phase | cif no. | Present in: | | |
|---|---|---|---|---|
| | | 3,1,12 | 2,2,12 | 1,3,12 |
| $RuO_2$ | | | Y | Y |
| $KRu_4O_8$ | | | | |
| $KRuO_4$ | | Y | | |
| KCl | | Y | Y | Y |

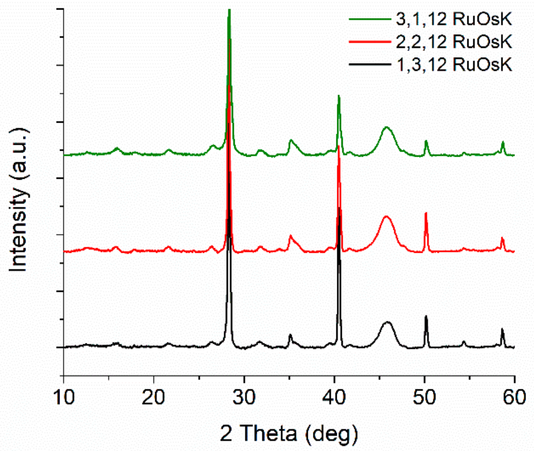


Figure S24. XRD patterns of (from bottom to top): 1,3,12 RuOsK, 2,2,12 RuOsK, and 3,1,12 RuOsK

| Phase | cif no. | Present in: | | |
|---|---|---|---|---|
| | | 3,1,12 | 2,2,12 | 1,3,12 |
| $RuO_2$ | | | | |
| $KRu_4O_8$ | | Y | Y | |
| $KRuO_4$ | | | | |
| KCl | | Y | Y | Y |

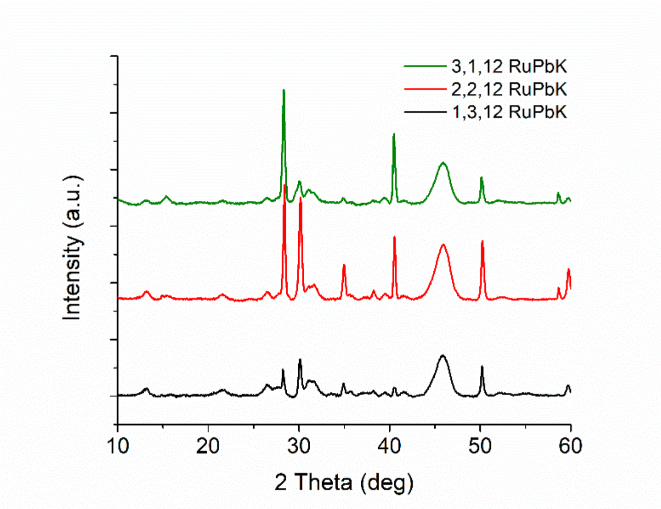


Figure S25. XRD patterns of (from bottom to top): 1,3,12 RuPbK, 2,2,12 RuPbK, and 3,1,12 RuPbK

| Phase | cif no. | Present in: | | |
|---|---|---|---|---|
| | | 3,1,12 | 2,2,12 | 1,3,12 |
| $RuO_2$ | | | | |
| $KRu_4O_8$ | | | | |
| $KRuO_4$ | | | | |
| KCl | | Y | Y | Y |
| $PbO_2$ | 96-900-8652 | Y | Y | Y |
| $Pb_3O_4$ | 96-901-2125 | | Y | Y |

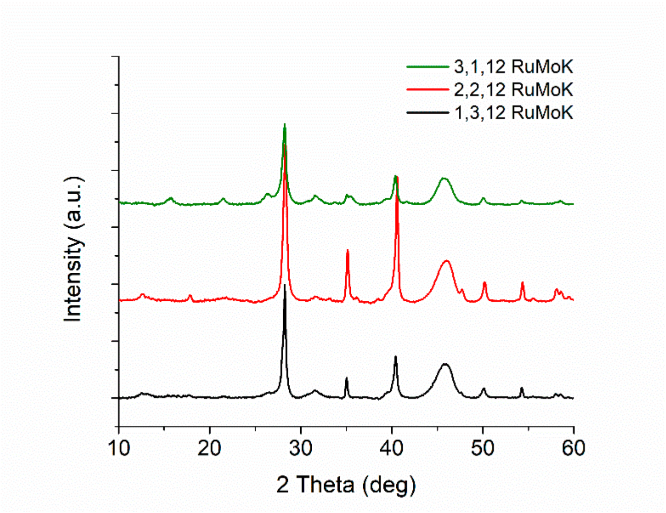


Figure S26. XRD patterns of (from bottom to top): 1,3,12 RuMoK, 2,2,12 RuMoK, and 3,1,12 RuMoK

| Phase | cif no. | Present in: | | |
|---|---|---|---|---|
| | | 3,1,12 | 2,2,12 | 1,3,12 |
| $RuO_2$ | | Y | Y | Y |
| $KRu_4O_8$ | | | | |
| $KRuO_4$ | | Y | | |
| KCl | | Y | Y | Y |

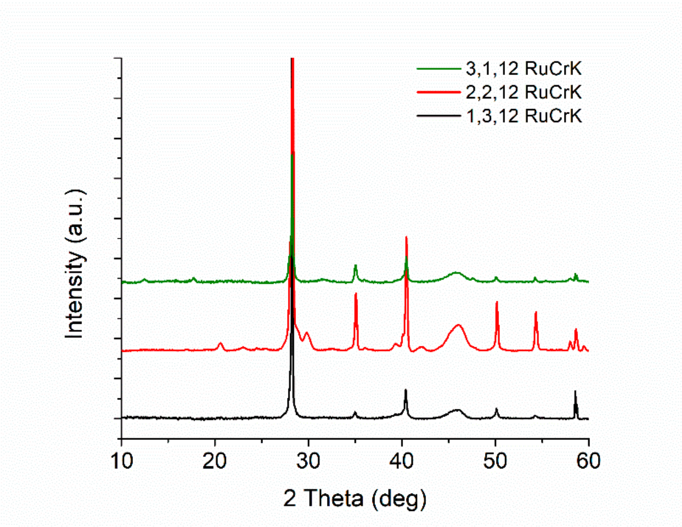


Figure S27. XRD patterns of (from bottom to top): 1,3,12 RuCrK, 2,2,12 RuCrK, and 3,1,12 RuCrK

| Phase | cif no. | Present in: | | |
|---|---|---|---|---|
| | | 3,1,12 | 2,2,12 | 1,3,12 |
| $RuO_2$ | | Y | Y | |
| $KRu_4O_8$ | | Y | | |
| $KRuO_4$ | | | | |
| KCl | | Y | Y | Y |
| $K_2CrO_4$ | 96-900-7571 | | Y | |

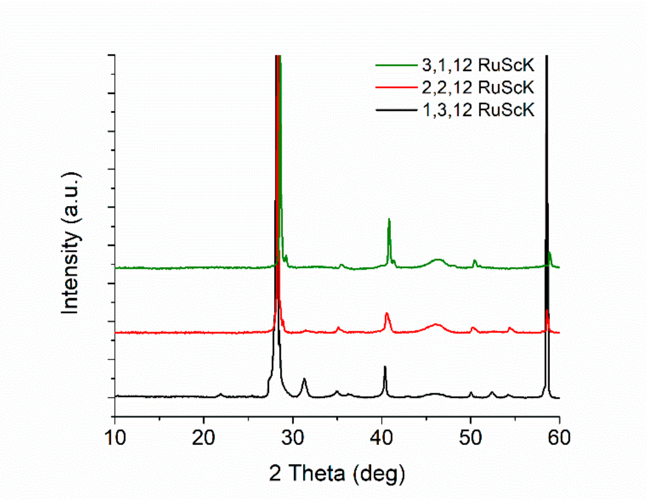


Figure S28. XRD patterns of (from bottom to top): 1,3,12 RuScK, 2,2,12 RuScK, and 3,1,12 RuScK

| Phase | cif no. | Present in: | | |
|---|---|---|---|---|
| | | 3,1,12 | 2,2,12 | 1,3,12 |
| $RuO_2$ | | Y | Y | Y |
| $KRu_4O_8$ | | Y | | |
| $KRuO_4$ | | | | |
| KCl | | Y | Y | Y |

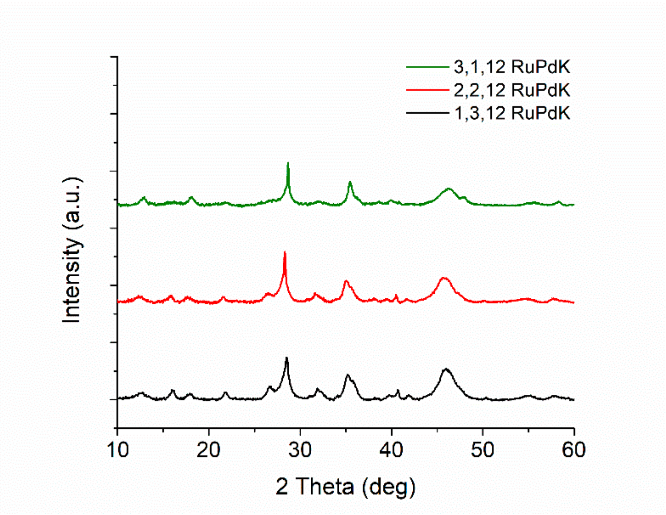


Figure S29. XRD patterns of (from bottom to top): 1,3,12 RuPdK, 2,2,12 RuPdK, and 3,1,12 RuPdK

| Phase | cif no. | Present in: | | |
|---|---|---|---|---|
| | | 3,1,12 | 2,2,12 | 1,3,12 |
| $RuO_2$ | | Y | Y | Y |
| $KRu_4O_8$ | | Y | Y | Y |
| $KRuO_4$ | | | | |
| KCl | | | Y | Y |
| $K_2PdCl_4$ | 96-101-0316 | Y | Y | Y |

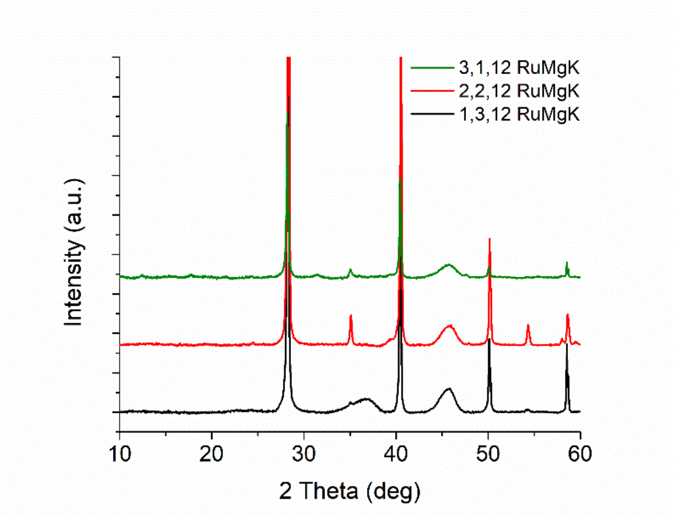


Figure S30. XRD patterns of (from bottom to top): 1,3,12 RuMgK, 2,2,12 RuMgK, and 3,1,12 RuMgK

| Phase | cif no. | Present in: | | |
|---|---|---|---|---|
| | | 3,1,12 | 2,2,12 | 1,3,12 |
| $RuO_2$ | | Y | Y | Y |
| $KRu_4O_8$ | | Y | | |
| $KRuO_4$ | | | | |
| KCl | | Y | Y | Y |

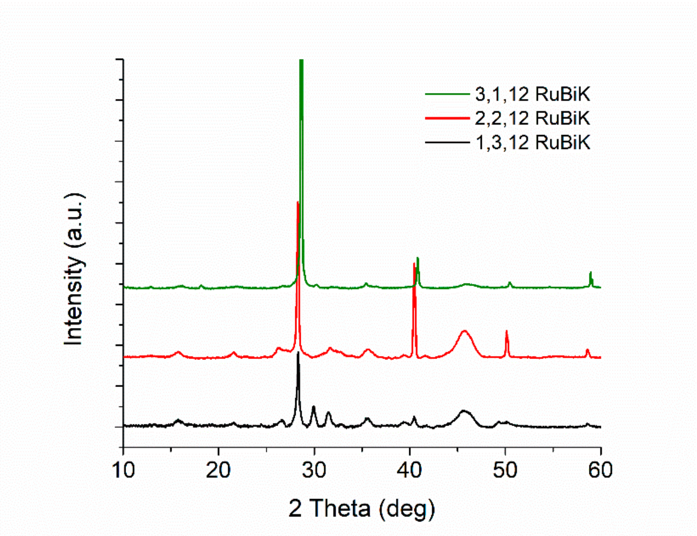


Figure S31. XRD patterns of (from bottom to top): 1,3,12 RuBiK, 2,2,12 RuBiK, and 3,1,12 RuBiK

| Phase | cif no. | Present in: | | |
|---|---|---|---|---|
| | | 3,1,12 | 2,2,12 | 1,3,12 |
| $RuO_2$ | | | | |
| $KRu_4O_8$ | | | | |
| $KRuO_4$ | | | | |
| KCl | | Y | Y | Y |
| $Bi_2O_3$ | 96-901-2328 | | | Y |

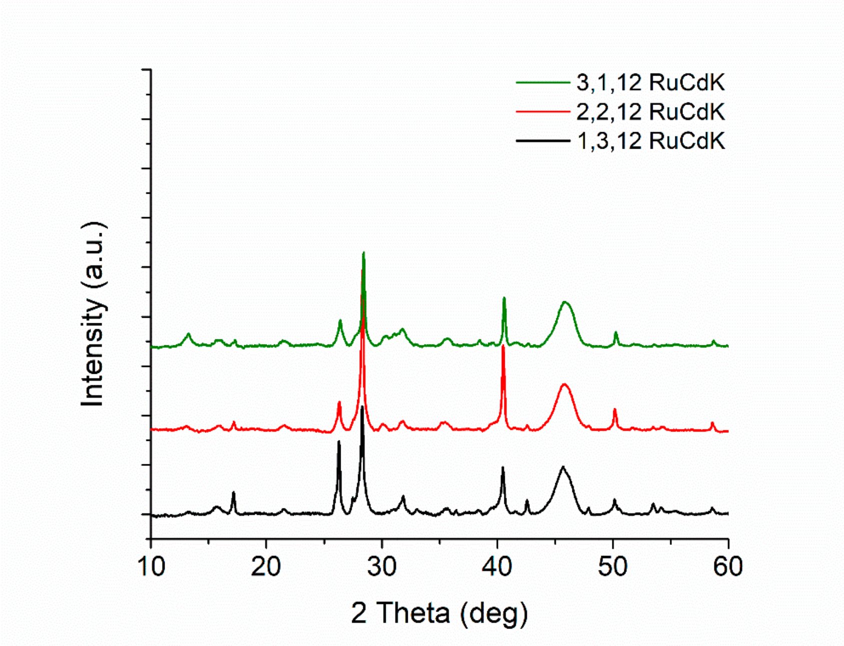


Figure S32. XRD patterns of (from bottom to top): 1,3,12 RuCdK, 2,2,12 RuCdK, and 3,1,12 RuCdK

| Phase | cif no. | Present in: | | |
|---|---|---|---|---|
| | | 3,1,12 | 2,2,12 | 1,3,12 |
| $RuO_2$ | | | | |
| $KRu_4O_8$ | | | | |
| $KRuO_4$ | | Y | Y | Y |
| KCl | | Y | Y | Y |
| CdO | 96-900-6686 | | | Y |

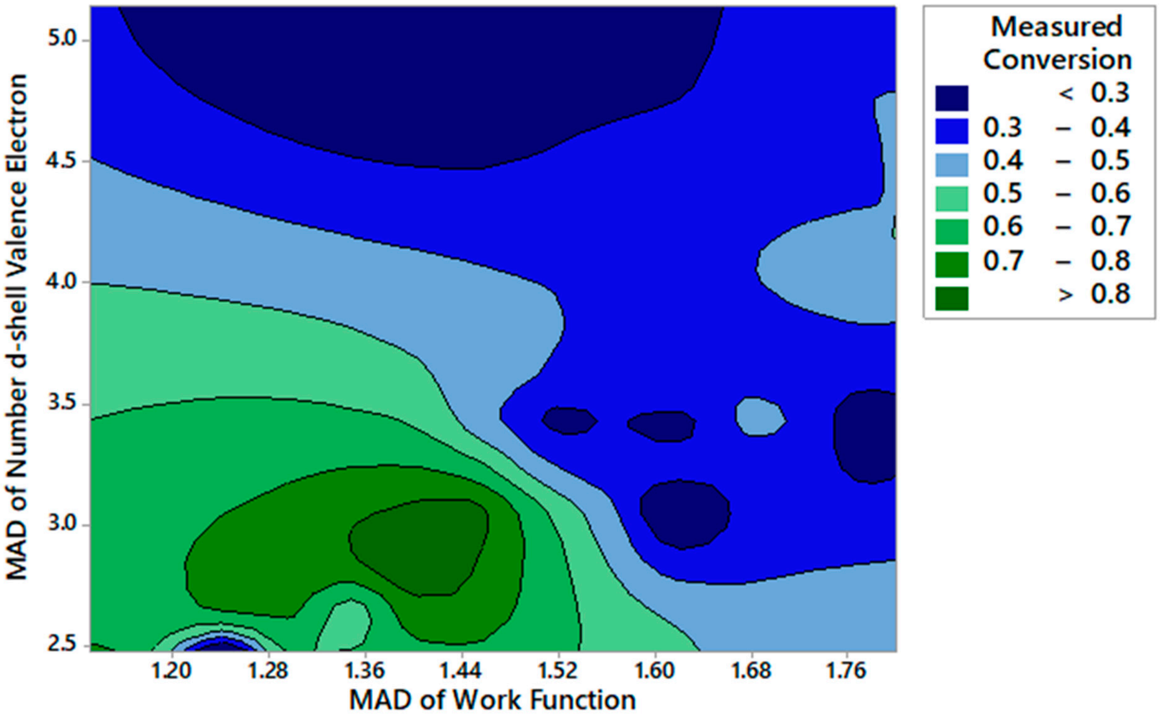


Figure S33. Ammonia decomposition activity at 300°C as a function of the mean absolute deviation (MAD) of the number of d-shell valence electrons and the MAD of the catalyst work function.
